# HSPB8 counteracts tumor activity of *BRAF*- and *NRAS*-mutant melanoma cells by modulation of RAS-prenylation and autophagy

**DOI:** 10.1038/s41419-022-05365-9

**Published:** 2022-11-18

**Authors:** Riccardo Cristofani, Margherita Piccolella, Marina Montagnani Marelli, Barbara Tedesco, Angelo Poletti, Roberta Manuela Moretti

**Affiliations:** 1 Dipartimento di Scienze Farmacologiche e Biomolecolari (DiSFeB), Università degli Studi di Milano, Milano, Italy; 2 Unit of Medical Genetics and Neurogenetics, Fondazione IRCCS Istituto Neurologico Carlo Besta, Milano, Italy

**Keywords:** Melanoma, Oncogenesis

## Abstract

Cutaneous melanoma is one of the most aggressive and lethal forms of skin cancer. Some specific driver mutations have been described in multiple oncogenes including *BRAF* and *NRAS* that are mutated in 60–70% and 15–20% of melanoma, respectively. The aim of this study was to evaluate the role of Small Heat Shock Protein B8 (HSPB8) on cell growth and migration of both BLM (BRAF^wt^/NRAS^Q61R^) and A375 (BRAF^V600E^/NRAS^wt^) human melanoma cell lines. HSPB8 is a member of the HSPB family of chaperones involved in protein quality control (PQC) system and contributes to chaperone assisted selective autophagy (CASA) as well as in the regulation of mitotic spindle. In cancer, HSPB8 has anti- or pro-tumoral action depending on tumor type. In melanoma cell lines characterized by low HSPB8 levels, we demonstrated that the restoration of HSPB8 expression causes cell growth arrest, reversion of EMT (Epithelial-Mesenchymal Transition)-like phenotype switching and antimigratory effect, independently from the cell mutational status. We demonstrated that HSPB8 regulates the levels of the active prenylated form of NRAS in *NRAS*-mutant and *NRAS*-wild-type melanoma cell lines. Consequently, the inhibition of NRAS impairs the activation of Akt/mTOR pathway inducing autophagy activation. Autophagy can play a dual role in regulating cell death and survival. We have therefore demonstrated that HSPB8-induced autophagy is a crucial event that counteracts cell growth in melanoma. Collectively, our results suggest that HSPB8 has an antitumoral action in melanoma cells characterized by *BRAF* and *NRAS* mutations.

## Introduction

Melanoma represents the most aggressive form of skin cancer. In its evolution to metastatic stage, it has a poor prognosis and high mortality rates despite many therapeutic options, which include surgery, radiotherapy, chemotherapy and immunotherapy. In addition, specific targeted therapies have been recently developed against somatic mutations in melanoma related genes regulating cell proliferation, migration, and apoptosis [1–3]. These mutations can occur in driver and mutually exclusive genes, such as *BRAF* and *NRAS*. Although other genes may be mutated in melanomas with concurrent *BRAF* or *NRAS* mutations, the melanoma classification falls in three-group based on the presence of: (i) mutant *BRAF*, (ii) mutant *NRAS*, (iii) or non-*BRAF*-mutant / non-*NRAS*-mutant [4]. About 50% of melanoma patients present mutations in *BRAF* gene which encodes a protein activating the mitogen-activated protein kinase (MAPK), which favors cell proliferation and survival. Among all *BRAF* mutations, most result in the substitution of the valine at position 600, with 90% represented by the BRAF^V600E^, and several minor V600 mutations (e.g., BRAF^V600K^, BRAF^V600D^, and BRAF^V600R^) [5]. The targeted therapies selectively inhibit BRAF^V600E^ activity in unresectable or metastatic melanoma (e.g., Vemurafenib and Dabrafenib) [6, 7].

RAS proteins belong to a superfamily of small GTPase proteins that regulate cell growth, survival, differentiation. The three RAS isoforms (NRAS, KRAS, and HRAS) are frequently mutated in cancer and constitutively activate intracellular signaling through a variety of pathways (mainly the MAPK and PI3K/Akt pathways) inducing cell-cycle dysregulation, pro-survival behavior, and cellular proliferation [8]. *NRAS*, the first oncogene identified in melanoma, is the one predominantly mutated among the RAS isoforms in melanoma (15–30% of cases) [9]. NRAS mutations occur at a glutamine in position 61 (NRAS^Q61R^, NRAS^Q61K^, NRAS^Q61L^) in 80% of the cases, while 6% of mutations involve amino acids at positions 12 and 13 (NRAS^G12V^, NRAS^G13R^) [10].

Targeted therapies for *NRAS*-mutant melanoma have been hampered by the high number of *NRAS* mutations and no one is available at present [11, 12]. Alternative therapeutic approaches have been proposed either to target the RAS/GTP interaction [13, 14] or to inhibit post-translational modification like prenylation or farnesylation, responsible for RAS insertion into the cell membrane [15]. To date, the targeted therapy for *NRAS*-mutant melanoma is based on MEK inhibitors, unfortunately with efficacy lower than that designed for *BRAF*-mutant melanoma [16, 17].

The Small Heat Shock Protein B8 (HSPB8) (also known as small stress protein-like protein (sHSP22) or protein kinase H11(H11)), is a member of the HSPB family of chaperones involved in the chaperone assisted selective autophagy (CASA) [18, 19]. HSPB8 exerts different activities [20], being involved in the protein quality control (PQC) in several neurodegenerative diseases [21–26] and in the regulation of mitotic spindle formation during mitosis [27, 28].

In cancer, HSPB8 exerts dual and opposite role depending on its expression levels and on cell type [27]. One relevant HSPB8 activity in cancer is linked to its pro-autophagic function mentioned above. Indeed, recently prognostic prediction model based on autophagy-related gene (ARG) signature studies conducted in a variety of tumors to correlate clinical stage and survival with autophagy genes indicated *HSPB8* as a prognostic ARG for tumor progression. In particular, HSPB8 overexpression correlates to high risk of recurrence and metastasis in breast cancer [29], hepatocarcinoma [30–33], and head and neck squamous cell carcinoma [34, 35], while low levels of HSPB8 are associated with negative prognosis in prostate cancer [36, 37].

The relevance of HSPB8 in melanoma has already been studied, but with conflicting results. Indeed, some investigators showed that HSPB8 is more expressed in melanoma cell lines and in patient biopsies compared to melanocytes and nevi [38], while others found that HSPB8 is downregulated by DNA hypermethylation in melanoma [39, 40].

Here, we elucidate the role of HSPB8 on cell growth, phenotype switching, and migration of human melanoma cell lines characterized by different driver mutations. We found that HSPB8 regulates RAS-prenylation both in *NRAS*-mutant and *NRAS*-wild-type melanoma cell lines, inhibiting the Akt/mTOR molecular pathway. Furthermore, we demonstrate that the HSPB8-mediated autophagy activation is a crucial event to prevent proliferation and migration of both *BRAF-* and *NRAS*-mutant melanoma.

## Materials and methods

### Antibodies and reagents

Mouse anti-HSPB8 (MAB4987) was from R&D System-Biotechne (Minneapolis, MN, USA). Rabbit anti-HSPB8 (PA5-76780) and rabbit anti-SQSTM1/p62 (PA5-20839) were from ThermoFisher Scientific (Waltham, MA, USA). Rabbit anti-E-cadherin (#3195), rabbit anti-N-cadherin (#13116), rabbit anti-vimentin (#5741), rabbit anti-ERK1/2 (#4695), rabbit anti-pERK1/2 (#4370), rabbit anti-cyclin D (#2926), rabbit anti-CDK4 (#12790), rabbit anti-Akt1 (#2938), rabbit anti-pAkt (Ser473) (#9271), rabbit anti-mTOR (#2983), rabbit anti-p-mTOR (Ser 2448) (#5536), rabbit anti-ATG5 (#12994) were from Cell Signaling (Danvers, MA, USA). Rabbit anti-BRAFV600E (RM8) was from RevMAb Biosciences (San Francisco, CA, USA). Mouse anti-pan-RAS (C-4) (sc-166691) was from Santa Cruz Biotechnology (Santa Cruz, CA, USA). Rabbit anti-LC3 (L8918) and mouse anti-alpha-tubulin (T6199) were from Sigma–Aldrich (St. Louis, MO, USA). Rabbit and mouse Horseradish-peroxidase-conjugated secondary antibody were from Cell Signaling. 3-Methyladenine (3-MA) (S2767) was from Selleckchem (Munich, Germany). Chloroquine (CQ) (C6628) was from Sigma–Aldrich.

### Cell culture

The human BLM (BRAF-wild-type) melanoma cell line was provided by Dr. G.N. van Muijen (Department of Pathology, Radbound University Nijmegen Medical Center, Nijmegen, The Netherlands). This cell line is a subline of BRO melanoma cells isolated from lung metastases after subcutaneous inoculation of nude mice with BRO cells [41]. We confirmed the NRAS^Q61R^ mutation by Sanger sequencing analysis.

The human A375 (BRAF^V600E^-mutant) melanoma cell line and MCF-7 breast cancer cell line were purchased from American Type Culture Collection (ATCC, Manassas, VA, USA). A375 cell line was authenticated using Short Tandem Repeat (STR) analysis (ATCC).

The human WM1552/5p (BRAF^V600E^-mutant) and WM115 (BRAF^V600D^-mutant) melanoma cell lines were provided by Dr. R. Giavazzi that obtained these cells from Dr. M. Herlyn (Wistar Institute, Philadelphia, PA) [42, 43]. The human IGR-39 (BRAF ^V600E^-mutant) melanoma cell line, kindly provided by Dr. C. La Porta, was obtained from Leibniz-Institut DSMZ-Deutsche Sammlung von Mikroorganismen and Zellkulturen GmbH (38124 Braunschweig, Germany). Primary human melanocytes were provided by Dr. F. Crovato (Regional Reference Centre for Human Epidermis in vitro Culture and Bank for Tissue Cryopreservation, Niguarda Hospital, Milano, Italy).

BLM, A375, IGR-39, WM1552/5p, WM115 cells were routinely cultured in DMEM supplemented with 10% FBS, glutamine (1 mmol/l) and antibiotics (100 IU/ml penicillin G sodium and 100 μg/ml streptomycin sulfate). MCF-7 cells were routinely cultured in RPMI 1640 medium supplemented with 10% FBS, glutamine (1 mmol/l) and antibiotics (100 IU/ml penicillin G sodium and 100 μg/ml streptomycin sulfate). Cells were cultured in humidified atmosphere of 5% CO_2_/95% air at 37 °C.

### RT-qPCR

Cells were plated at 5 × 10^4^ cells/well in six-well multiwell plates and after 48 h were harvested and centrifuged 5 min at 100 × *g* at 4 °C; pellets were resuspended in 600 μL of TRI Reagent (#T9424; Sigma–Aldrich) and total RNA isolated according to manufacturer’s instructions. RNA quantification was carried out by absorbance at 260 nm. One microgram of total RNA was treated with DNAse I (AMPD1; Sigma–Aldrich), and reverse transcribed into cDNA using the High-Capacity cDNA Reverse Transcription Kit (4368813; Life Technologies) according to the manufacturer’s protocol. All primers for real-time PCR were designed using the program Primer 3.

The primers were synthesized by MWG Biotech (Ebersberg, Germany) with the following sequence:

*HSPB8*_F: 5’-ATACGTGGAAGTTTCAGGCA-3’

*HSPB8*_R: 5’-TCTCCAAAGGGTGAGTACGG-3’

*SQSTM1/p62*_F: 5’- CCAGAGAGTTCCAGCACAGA-3’

*SQSTM1/p62*_R 5’- CCGACTCCATCTGTTCCTCA-3’

The evaluated efficiency of each set of primers was close to 100% for both target and reference gene. Real-time PCR was performed using the CFX 96 Real-Time System (Bio-Rad) in a 10 μL total volume, using the iTaq SYBR Green Supermix (Bio-Rad), and with 500 nM primers.

PCR cycling conditions were as follows: 94 °C for 10 min, 40 cycles at 94 °C for 15 sec and 60 °C for 1 min.

Melting curve analysis was performed at the end of each PCR assay as a control for specificity. Data were analyzed and values were normalized to those of RPLP0:

*RPLP0*_F: 5’-GTGGGAGCAGACAATGTGGG-3’

*RPLP0*_R: 5’-TGCGCATCATGGTGTTCTTG-3’

All statistics were performed with ∆C_t_ values.

### Mutational analysis

To analyze the presence of the *NRAS* mutation in BLM cells we have amplified NRAS using the following primers:

*NRAS*_F: 5’-AAGTCAGGACCAGGGTGTCA-3’

*NRAS*_R: 5’-CCGGGGTCTCCAACATTTTTC-3’

PCR cycling conditions were as follows:

5 min at 95 °C, 35 cycles at: 95 °C 30 sec, 57 °C 30 sec, 72 °C 60 sec, 5 min at 72 °C.

The amplification product was separated by agarose gel electrophoresis and the 725 bp amplicon was sequenced by Sanger Methods (Eurofins Genomics).

The results of sequencing were processed by Snap Gene Viewer 5.0.4 software.

### Transient overexpression of HSPB8

BLM and A375 cells were seeded in six-well plates at 5 × 10^4^ cells/well. After 24 h, cells were transfected with 1 µg of pcDNA3.1 (mock) or pCI-HSPB8, pCI-HSPB8^K141E^, and pCI-HSPB8^K141N^ [44] by Lipofectamine 3000 (L300001) (Invitrogen-ThermoFisher Scientific) according to the manufacturer’s instructions. Transfection was performed for 24, 48, and 72 h.

### siRNA *ATG5*

To silence endogenous ATG5 expression, BLM and A375 cells were transfected with 20 nM negative control siRNA (NC siRNA) (#6568) and 20 nM *ATG5* siRNA I (#6345) (Cell Signaling) by Lipofectamine RNAi max Reagent (13778) (Invitrogen-ThermoFisher Scientific) according to the manufacturer’s instructions. Transfection was performed for 72 h.

### Cell proliferation assay

BLM and A375 cells were plated at the density of 5 × 10^4^ cells/well in six-well plates. After 24 and 48 h of overexpression of HSPB8 protein (wild-type or mutated) the cells were harvested and counted by hemocytometer. Each proliferation assay was repeated three times.

### MTT viability assay

BLM and A375 cells were seeded at a density of 3 × 10^4^ cells/well in 24-well plates. After 24 h or 48 h of mock or HSPB8 overexpression the medium was changed with MTT (3-(4,5 dimethylthiazol-2-yl)-2,5-diphenyl tetrazolium bromide) solution (0.5 mg/ml) in DMEM without phenol red and FBS; cells were incubated at 37 °C for 30 min and formazan precipitate was dissolved with isopropanol. Absorbance at 550 nm was measured through an EnSpire Multimode Plate reader (PerkinElmer, Milano, Italy).

### Western blotting

For Western Blot (WB) studies cells were seeded in 6 cm dishes at the density of 8 × 10^4^ cells/dish. At the end of experiments, cells were washed with PBS and lysed with RIPA buffer (0.05 mol/L Tris HCl pH 7.7, 0.15 mol/L NaCl, 0.8% SDS, 10 mmol/L EDTA, 100 μM/L NaVO_4_, 50 mmol/L NaF, 0.3 mmol/L PMSF, 5 mmol/L iodoacetic acid) containing leupeptin (50 μg/ml,), aprotinin (5 μl/ml), and pepstatin (50 μg/ml). Protein extract (EP) concentration was determined using BCA protein assay kit (ThermoFisher Scientific). Fifteen to thirty-five micrograms of EP was separated through SDS gel electrophoresis and transferred to PVDF (for LC3 analysis) or nitrocellulose membranes. After blocking with non-fat dried milk, membranes were incubated at 4 °C overnight using the specific antibodies: mouse anti-HSPB8 (1:1000), rabbit anti-HSPB8 (1:1000), rabbit anti-E-cadherin (1:1000), rabbit anti-N-cadherin (1:1000), rabbit anti-vimentin (1:1000), rabbit anti-ERK1/2 (1:1000), rabbit anti-pERK (1:1000), rabbit anti-cyclin D1 (1:1000), rabbit anti-Akt1 (1:1000), rabbit anti-pAkt (Ser473) (1:1000), rabbit anti-mTOR (1:1000), rabbit anti-p-mTOR (1:1000), rabbit anti-BRAF^V600E^ (1:1000), mouse anti-pan-RAS (1:1000), rabbit anti-LC3 (1:1000), rabbit anti-SQSTM1/p62 (1:3000), rabbit anti-ATG5 (1:1000), mouse anti-alpha-tubulin (1:2000).

Peroxidase-conjugated secondary anti-rabbit or anti-mouse antibodies were used for 1 h at room temperature and the membranes were processed using enhanced chemiluminescence kit Cyanagen Ultra (Cyanagen, Bologna, Italy).

In each WB experiment tubulin expression was evaluated as a loading control.

Uncropped WB images are available in Supplementary materials.

### Migration assay

Cell migration assay was performed using a 48-well-Boyden chamber (NeuroProbe, Inc., Gaithersburg, MD, USA) containing 8 µm polycarbonate membranes (Nucleopore, Concorezzo, Milan, Italy). Membranes were coated on one side with 50 µg/ml laminin or 50 µg/ml fibronectin rinsed once with PBS, and then placed in contact with the lower chamber containing DMEM medium. BLM and A375 cells were transfected with pCI-hHSPB8. At the end of transfection, the cells were collected and added (10^5^ cells) to the top of each chamber and allowed to migrate through coated filters for 4 h. The migrated cells attached on the lower membrane surfaces were fixed, stained with Diffquik (Biomap, Italy) and counted in standard optical microscopy (20X).

The results of three separate experiments of migration are presented. Each experimental group consisted of 12 samples.

### Membrane-bound RAS prenylated

To measure membrane-bound RAS prenylated, cells were harvested in PBS buffer, sonicated and centrifugated at 800 × *g* for 10 min at 4 °C. The supernatants were collected and centrifugated at 18,000 × *g* for 30 min at 4 °C. The pellets containing plasma membrane fractions (Mb) were collected and resuspended in RIPA buffer. Protein concentration of Mb was determined using BCA protein assay kit (ThermoFisher Scientific). Ten micrograms of Mb was separated through SDS gel electrophoresis.

### Immunofluorescence

For immunofluorescence studies the cells were seeded at 3 × 10^4^ cells/well on polylysine-coated coverslips in 24-well plates. After 48 h, cells were fixed with 3% paraformaldehyde / 2% sucrose. Cells were washed with PBS and permeabilized with 0.1% Triton X-100 in PBS for 20 min followed by incubation in blocking solution (1% horse serum in PBS) for 1 h. Cells were incubated with the following antibodies diluted in PBS with 3% BSA overnight at 4 °C: mouse anti-HSPB8 (1:300), rabbit anti-LC3 (1:500). The cells were washed with PBS and incubated with secondary antibodies conjugated with TRITC AlexaFluor 594 for detection of HSPB8 (1:1000) or FITC AlexaFluor-488 for detection of LC3 (1:2000). Nuclei were stained with Hoechst (dilution 1:10,000). Labeled cells were examined under Zeiss Axiovert 200 microscope (Zeiss, Oberkochen, Germany) with ×40 or ×63/1.4 objective lens linked to a Coolsnap Es CCD camera (Ropper Scientific-Trenton, NJ, USA).

Cell morphology was analyzed by phase-contrast microscopy (PC) or differential interference contrast microscopy (DIC).

### Statistical analysis

All experiments were performed three times and the results were analyzed by unpaired Student’s *t*-test or by one-way analysis of variance ANOVA, followed by Dunnet or Bonferroni post-test using the PRISM software (GraphPad Software, La Jolla, Ca, USA).

## Results

### Effect of HSPB8 overexpression on growth and viability in BLM and A375 cells

In this study, we investigated HSPB8 protein expression and its role in melanoma cell lines carrying different mutations.

**Fig. 1:**
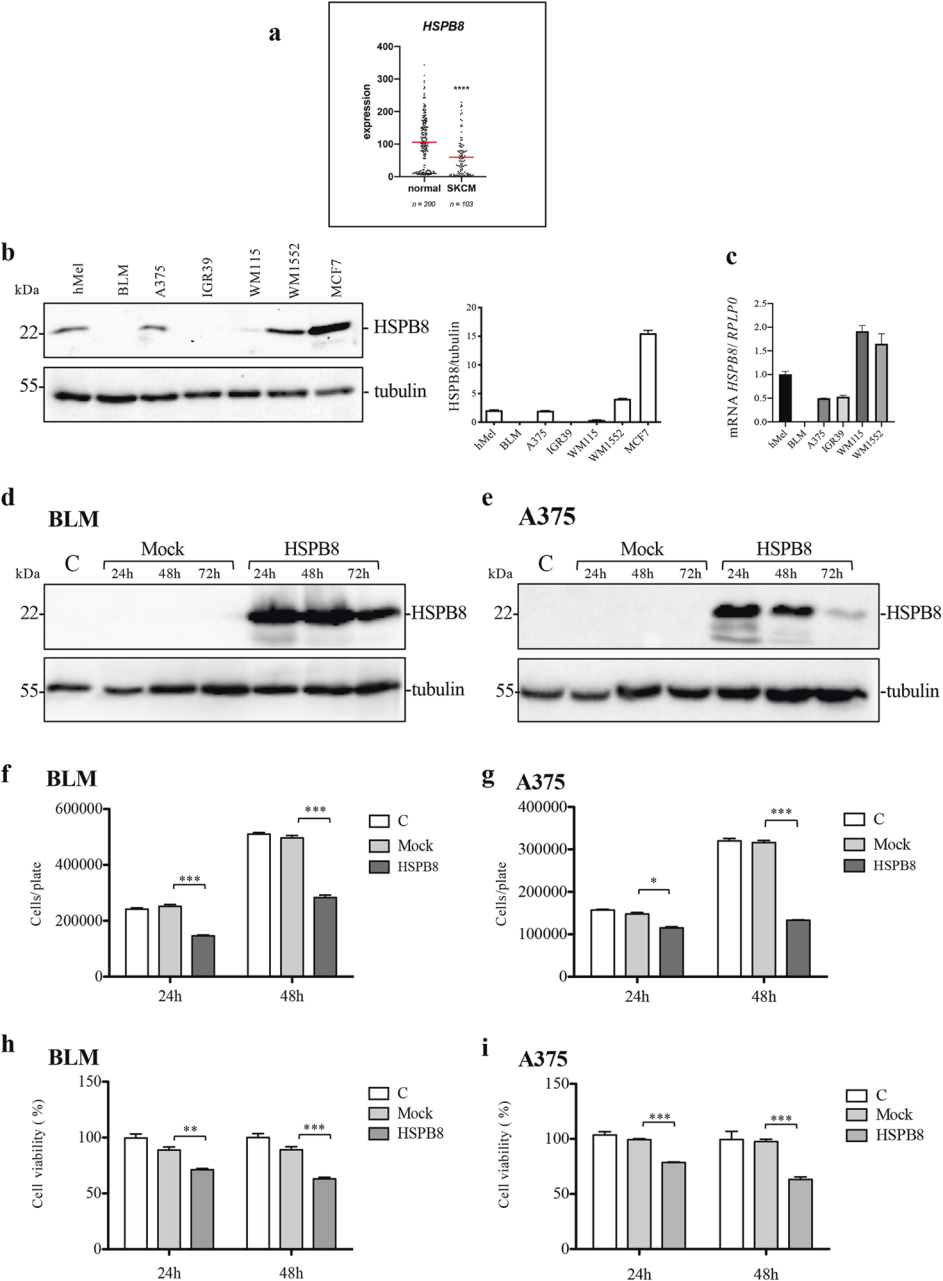
Expression of HSPB8 in melanoma cell lines. **a** Analysis of *HSPB8* gene expression in skin cutaneous melanoma (SKCM) vs normal tissue (skin) as reported in OncoDB database. Scatter plot represents the mean of *HSPB8* expression value (red line) obtained from RNA-Seq data normalized using Transcripts Per Million (TPM). Student’s *t*-test (****p < 0.0001) **b** HSPB8 expression levels in different melanoma cell lines compared with human melanocytes and MCF-7 breast cancer cells. Tubulin was used as loading control. Three independent biological samples for each condition were analyzed (*N* = 3), bar graph represents the mean optical density ± SD. **c** *HSPB8* mRNA expression in different melanoma cell lines analyzed by RT-qPCR. Data have been normalized to the amount of *RPLP0* mRNA. Bar graph represents the mean of four independent biological samples (*N* = 4) ±SD. **d**, **e** HSPB8 WB analysis of BLM (**d**) and A375 (**e**) cells overexpressing HSPB8. **f**, **g** Cell count assay was performed in BLM (**f**) and A375 (**g**) cells overexpressing HSPB8. Four independent biological samples for each condition were analyzed (*N* = 4), bar graph represents the mean relative cell count ± SD. Statistical analysis was performed using Student’s *t*-test (*p < 0.05 vs. mock 24 h; ***p < 0.001 vs. mock 24 h and 48 h). **h**, **i** Cell viability assay was performed in BLM (**h**) and A375 (**i**) cells overexpressing HSPB8. Six independent biological samples for each condition were analyzed (*N* = 6), bar graph represents the mean relative cell viability ± SD. Statistical analysis was performed using Student’s *t*-test (**p < 0.01 vs. mock 24 h; ***p < 0.001 vs. mock 24 h and 48 h).

We initially analyzed *HSPB8* gene expression in 103 samples of skin cutaneous melanoma patients of the TCGA study as well as 200 normal tissue (skin) of the GTEx studies available in OncoDB database [45]. The comparison between skin cutaneous melanoma and matched normal samples clearly shows that human *HSPB8* gene expression is downregulated in skin cutaneous melanoma (Fig. 1a).

We then analyzed HSPB8 expression levels in different melanoma cell lines compared with human melanocytes and the MCF-7 breast cancer cells highly enriched in HSPB8 [46, 47].

The analysis was carried out in a panel of human melanoma cell lines: BLM (NRAS^Q61R^-mutant), A375 (BRAF^V600E^-mutant), IGR-39, WM1552 (BRAF^V600E^-mutant), and WM115 (BRAF^V600D^-mutant). WB analysis (Fig. 1b) shows that HSPB8 protein level is variable among melanoma cell lines, but always lower than in MCF-7 cells.

Therefore, we assessed whether the different HSPB8 protein levels observed in melanoma cells were due to regulation of transcription or translation/stability by analyzing its mRNA level by RT-qPCR (Fig. 1c) demonstrating that HSPB8 mRNA levels recapitulate that of its protein levels found in WB. Only, WM115 cells show very low HSPB8 protein level with very high mRNA level. Due to the marked heterogeneity of HSPB8 expression profile, to better understand the role of HSPB8 we selected the BLM and A375 cell lines characterized by low or very low HSPB8 levels, respectively.

Exogenous HSPB8 was thus overexpressed in BLM cells for 24, 48, and 72 h. High levels of HSPB8 were found already at 24 h and the protein remained stable up to 72 h (Fig. 1d). The same experimental approach was applied to A375 cells. WB analysis shows that HSPB8 expression was maximum 24 and 48 h after transfection (Fig. 1e). Notably, HSPB8 overexpression induced a significant decrease of cell proliferation both in BLM and A375 cell lines (Fig. 1f, g).

To better characterize the antiproliferative effect, we analyzed the effect of HSPB8 overexpression on cell viability by MTT assays. The results shown in Fig. 1h, i demonstrate that HSPB8 exerts a cytotoxic effect in both cell lines.

Therefore, HSPB8 exerts an antitumoral action both in BLM and A375 cells.

### Effect of HSPB8 overexpression on cell phenotype and motility in BLM and A375 cells

Malignant melanomas are characterized by a high invasive and metastatic behaviors. These characteristics are often linked to a switching of the cellular phenotype. This phenomenon, that resembles the Epithelial-Mesenchymal Transition (EMT), involves the decrease of E-cadherin, the increase of mesenchymal proteins (N-cadherin, vimentin) and causes a migratory and invasive phenotype in melanomas [48, 49].

**Fig. 2:**
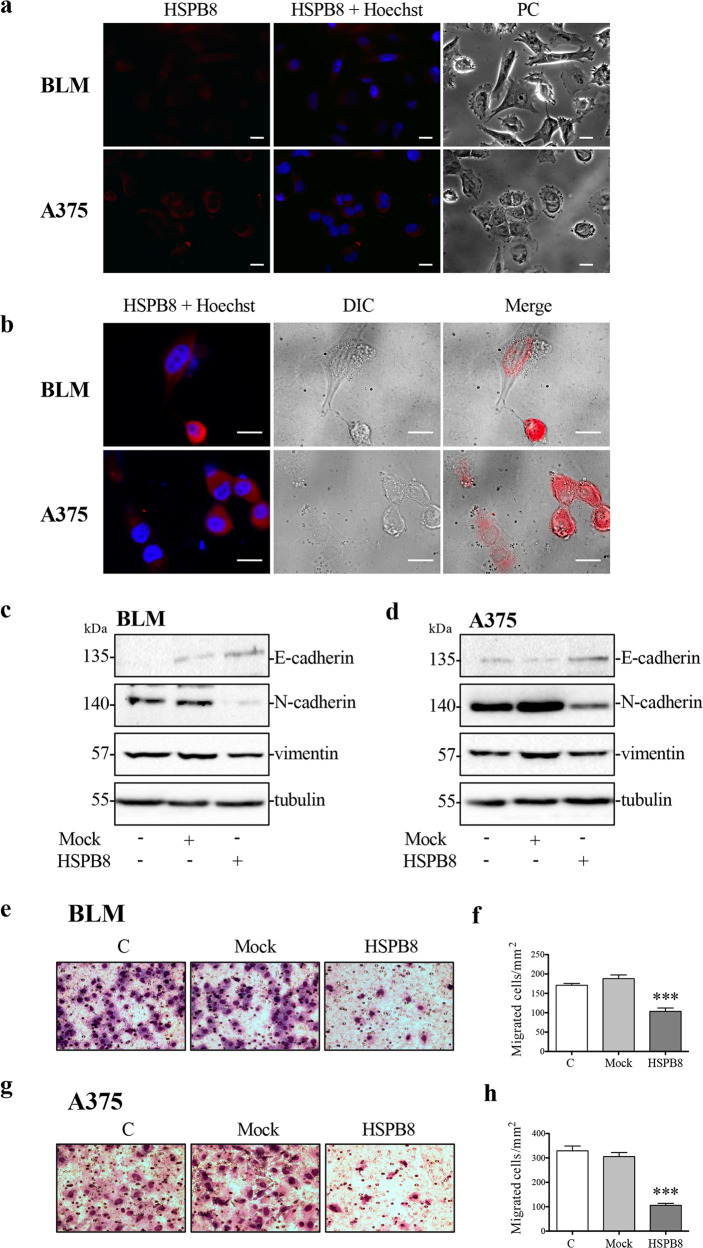
Effect of HSPB8 overexpression on cell phenotype and motility in BLM and A375. **a** Immunofluorescence analysis of HSPB8 espression in BLM and A375 cells. Nuclei were stained by Hoechst. Cell morphology was analyzed by phase-contrast microscopy (PC) Scale bar, 20 μm. **b** HSPB8 expression (red) was carried out by immunofluorescence in BLM and A375 cells overexpressing HSPB8 (48 h). Nuclei were stained by Hoechst. Cell morphology was analyzed by Differential Interference Contrast microscopy (DIC). Scale bar, 20 μm. **c**, **d** WB analysis of E-cadherin, N-cadherin, and vimentin in BLM (**c**) and A375 (**d**) cells overexpressing HSPB8 (48 h). Tubulin was used as loading control. **e**, **f** Cellular motility of BLM cells was analyzed by Boyden’s chamber assay. Control (C), mock and HSPB8 overexpressed cells (for 48 h) were analyzed in migratory assays on laminin-coated membranes. Representative images of migrated cells in each condition are shown (**e**). The graph reports the quantification of migrated cells/mm^2^. One-way Anova followed by Bonferroni post-hoc test (***p < 0.001 vs. C and mock) was performed. (*N* = 12). (**f**). **g**, **h** Cellular motility of A375 cells was analyzed by Boyden’s chamber assay. Cells were analyzed in migratory assays on laminin-coated membranes 48 h after transfection. Representative images of migrated cells in each condition are shown (**g**). The graph reports the quantification of migrated cells/mm2. One-way ANOVA followed by Bonferroni post-hoc test was performed (***p < 0.001 vs. C and mock). (*N* = 12) (**h**).

We then analyzed BLM and A375 cell lines phenotype in basal conditions of endogenous HSPB8 (Fig. 2a). Phase-contrast microscopy (PC) images show that BLM cells present a mesenchymal-like morphology, while A375 cells show an apparent epithelial-like phenotype. Morphological changes characterized by cellular rounding occurs in cells that overexpressed HSPB8 compared to cells with low levels of HSPB8 in both cellular models (Fig. 2b). We then analyzed this phenotype switching by evaluating the expression of EMT markers. The WBs show that both cell lines express low levels of E-cadherin and high levels of N-cadherin and vimentin, compatibly with mesenchymal-like phenotypes. HSPB8 overexpression induces a reversion of EMT-like phenotype increasing E-cadherin levels and reducing N-cadherin and vimentin expression in BLM and A375 cells respectively (Fig. 2c, d).

Since the reversion of EMT-like phenotype may modulate migration of tumoral cells, we analyzed the effect of HSPB8 overexpression in BLM and A375 cell migration by Boyden’s chamber.

Preliminary experiments demonstrated that both cell lines migrate similarly on both laminin and fibronectin (data not shown), so we conducted migration assay using laminin-coated membrane. The HSPB8 overexpression significantly reduced BLM (Fig. 2e, f) and A375 (Fig. 2g, h) migration compared to control and mock cells.

Taken together, these results show that the loss of HSPB8 contributes to melanoma transformation and its re-expression can reduce tumor aggressiveness acting on phenotype switching and migratory capacity, regardless of the mutational status of cells.

### Effect of HSPB8 overexpression on RAS expression and activity in BLM and A375 cells

To deeply investigate the molecular mechanisms responsible for HSPB8 antitumoral activity, we analyzed the effect of HSPB8 overexpression on the signaling pathways controlled by NRAS and BRAF.

In BLM cells, NRAS pathway is constitutively active due to NRAS^Q61R^ mutation resulting in MAPK and Akt/mTOR pathways activation.

First, we analyzed the effect of HSPB8 overexpression on RAS expression.

**Fig. 3:**
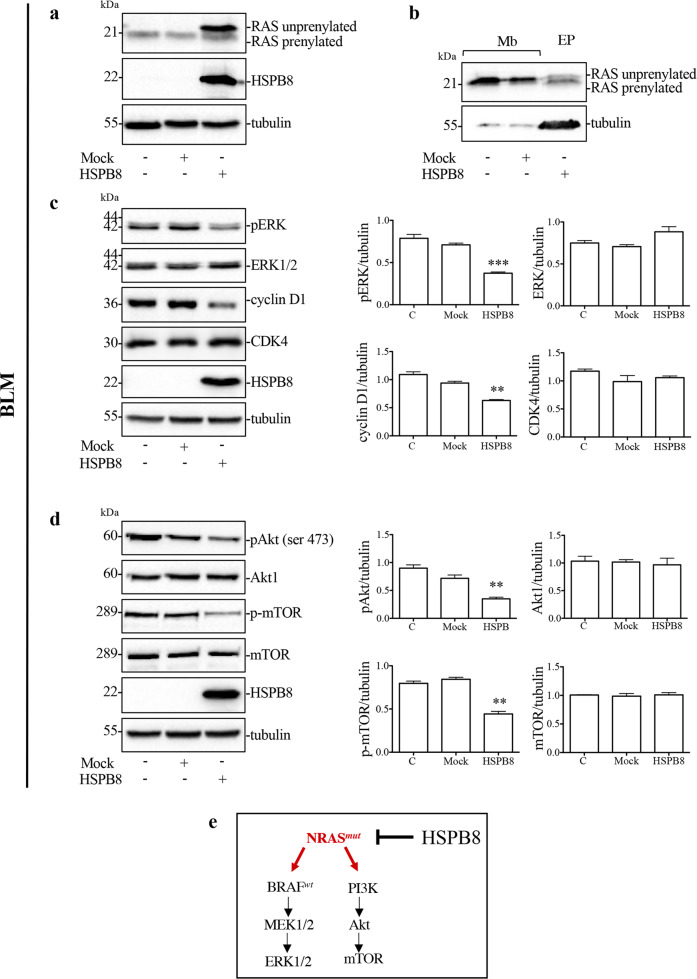
Effect of HSPB8 overexpression on NRAS pathway in BLM cells. **a** WB analysis of RAS protein level in BLM cells overexpressing HSPB8 (48 h). Tubulin was used as loading control. **b** WB analysis of RAS protein levels in plasma membranes (Mb) of BLM cells expressing HSPB8 (48 h) compared with total protein extracts (EP). **c** Effect of HSPB8 overexpression (48 h) on MAPK pathway. Tubulin was used as loading control. Three independent biological samples for each condition were analyzed (*N* = 3), bar graph represents the mean optical density ± SD. Statistical analysis was performed using One-way ANOVA followed by Bonferroni post-hoc test (**p < 0.01 vs. C and mock ***p < 0.001 vs. C and mock). **d** Effect of HSPB8 overexpression (48 h) on Akt/mTOR pathway. Tubulin was used as loading control. Three independent biological samples for each condition were analyzed (*N* = 3), bar graph represents the mean optical density ± SD. Statistical analysis was performed using One-way ANOVA followed by Bonferroni post-hoc test (**p < 0.01 vs. C and mock). **e** Schematic representation of HSPB8 effect on NRAS^mut^ activity.

The results in Fig. 3a show an increase of RAS expression with a clear upshift in samples overexpressing HSPB8. This might be due to a lower apparent molecular weight of prenylated RAS, since the unprenylated RAS form is characterized by a reduced mobility in SDS-PAGE compared to prenylated RAS. Prenylated active form of RAS is more hydrophobic and localizes in plasma membrane. The analysis of RAS levels in plasma membranes (Mb) after HSPB8 overexpression highlights the reduction of the prenylated form of RAS (Fig. 3b), indicating a decreased RAS activity in BLM cells.

We analyzed the main factors involved in the MAPK signaling pathway constitutively activated by NRAS mutation, analyzing pERK, ERK, cyclin D1, and CDK4 expression. Results show that HSPB8 overexpression reduced ERK phosphorylation and decreased the expression of cyclin D1, while CDK4 was unchanged (Fig. 3c). This confirms the ability HSPB8 to reduce NRAS activity and consequently the activation/expression of proteins that stimulate cell-cycle progression. Since NRAS regulates Akt/mTOR signaling activation, we analyzed the Akt and mTOR activity demonstrating that HSPB8 overexpression reduced their phosphorylation (Fig. 3d).

These results suggest that HSPB8 modulates MAPK and Akt/mTOR pathways (Fig. 3e) acting on RAS-prenylation.

**Fig. 4:**
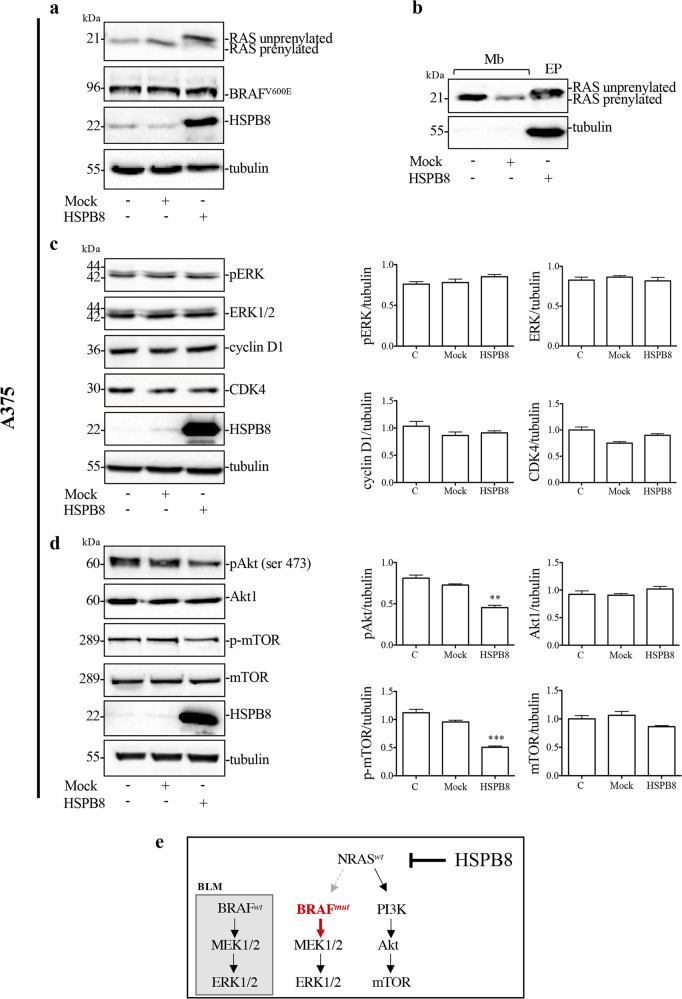
Effect of HSPB8 overexpression on NRAS pathway in A375 cells. **a** WB analysis of RAS and BRAF^V600E^ protein levels in A375 cells overexpressing HSPB8 (48 h). Tubulin was used as loading control. **b** WB analysis of RAS protein levels in plasma membranes (Mb) of BLM cells expressing HSPB8 (48 h) compared with total protein extracts (EP). **c** Effect of HSPB8 overexpression (48 h) on MAPK pathway. Tubulin was used as loading control. Three independent biological samples for each condition were analyzed (*N* = 3), bar graph represents the mean optical density ± SD. Statistical analysis was performed using One-way ANOVA. **d** Effect of HSPB8 overexpression (48 h) on Akt/mTOR pathway. Tubulin was used as loading control. Three independent biological samples for each condition were analyzed (*N* = 3), bar graph represents the mean optical density ± SD. Statistical analysis was performed using One-way ANOVA followed by Bonferroni post-hoc test (**p < 0.01 vs. C and mock ***p < 0.001 vs. C and mock). **e** Schematic representation of HSPB8 effect on NRAS^*wt*^ and BRAF^*mut*^ activity.

Similarly, experiments performed on A375 cells BRAF^V600E^-mutant, which determines a constitutive activation of MAPK pathway, show that HSPB8 can increase unprenylated RAS (Fig. 4a) reducing its active form localized in Mb (Fig. 4b) without modifying BRAF^V600E^ expression (Fig. 4a). In A375 cells, HSPB8 overexpression did not modify ERK phosphorylation and cyclin D1 expression (Fig. 4c), whilst it reduced both Akt and mTOR phosphorylation (Fig. 4d) as observed in BLM cells. In A375 cells, HSPB8 possibly acts on NRAS^*WT*^ while BRAF mutation hampers the effects of HSPB8 on MAPK pathway, differently from BLM cells where BRAF is wild-type (Fig. 4e).

Collectively, these results demonstrate that HSPB8 acts both on mutated and wild-type NRAS proteins, and this event is crucial to explain antitumoral effects in melanoma cell lines.

### Effect of HSPB8^K141E^ and HSPB8^K141N^ overexpression on RAS expression and activity in BLM and A375 cells

**Fig. 5:**
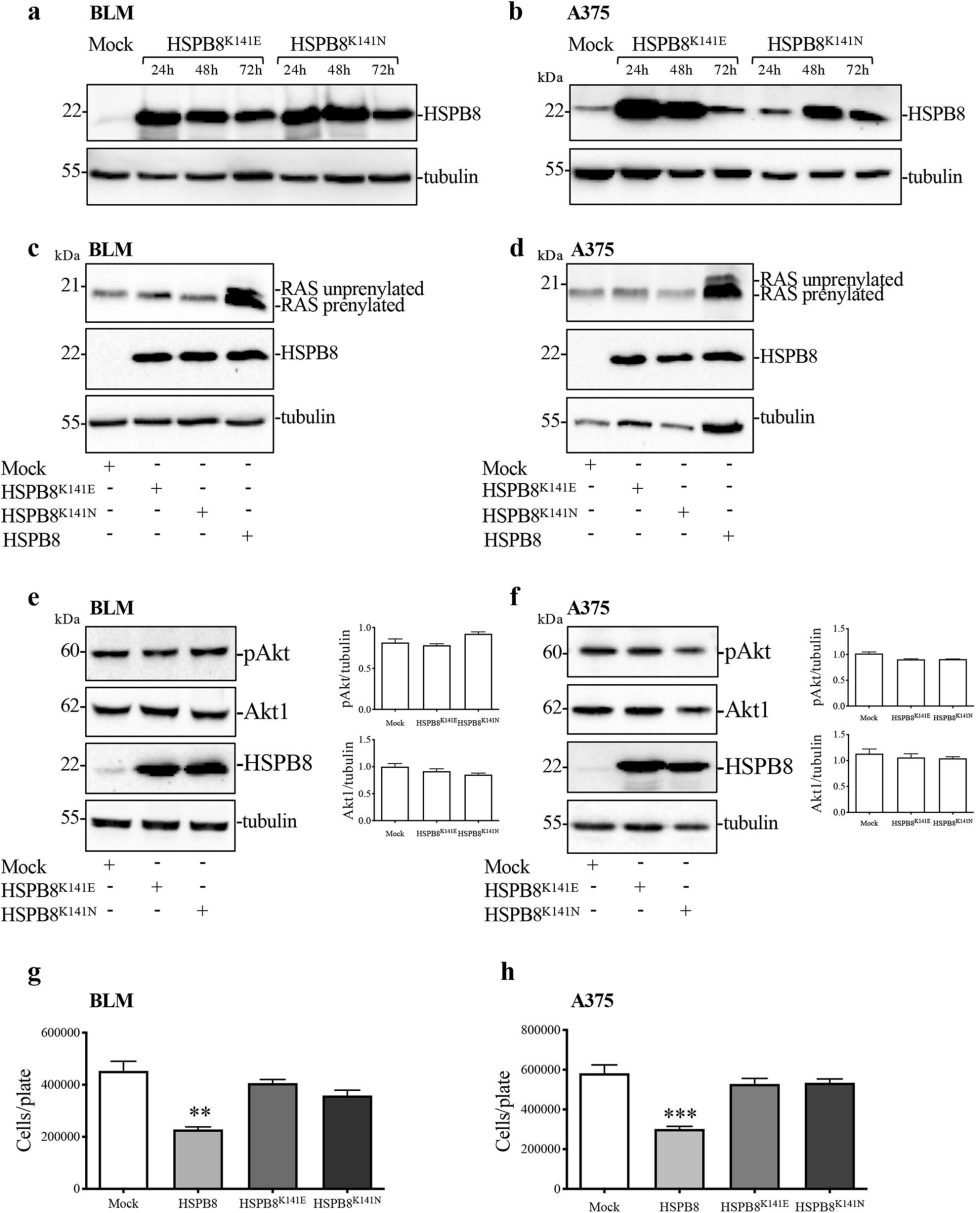
Effect of HSPB8^K141E^ and HSPB8^K141N^ overexpression on NRAS pathway in BLM and A375 cells. **a**, **b** WB analysis of HSPB8 in BLM (**a**) and A375 (**b**) cells overexpressing HSPB8^K141E^ and HSPB8^K141N^ for 24, 48, and 72 h. **c**, **d** WB analysis of RAS protein levels in BLM (**c**) and A375 cells (**d**) overexpressing HSPB8^K141E^, HSPB8^K141N^, and HSPB8 (48 h). **e**, **f** Effect of mutant HSPB8 overexpression (48 h) on Akt and p-Akt in BLM (**e**) and A375 (**f**) cells. Tubulin was used as loading control. Three independent biological samples for each condition were analyzed (*N* = 3), bar graph represents the mean optical density ± SD. **g**, **h** Cell count assay was performed in BLM (**g**) and A375 cells (**h**) expressing mutant HSPB8 (48 h). Four independent biological samples for each condition were analyzed (*N* = 4), bar graph represents the mean relative cell viability ± SD. Statistical analysis was performed using Student’s *t*-test (**p < 0.01 vs. mock; (***p < 0.001 vs. mock).

In order to gain information on the molecular mechanism by which HSPB8 exerts its action in melanoma, we transiently transfected mutated forms of HSPB8 (HSPB8^K141E^ and HSPB8^K141N^) in BLM (Fig. 5a) and A375 cells (Fig. 5b). HSPB8^K141E^ and HSPB8^K141N^ are characterized by an altered capability to dimerize and to interact with the HSPB8 co-chaperone Bcl2-Associated Athanogene 3 (BAG3) [19]. The results show that the overexpression of the two HSPB8 mutants prevented the increase of unprenylated NRAS (Fig. 5c, d) and the reduction of Akt phosphorylation both in BLM and A375 cells (Fig. 5e, f). Moreover, HSPB8^K141N^ and HSPB8^K141E^ expression was unable to reduce BLM and A375 cell proliferation as observed in HSPB8^*WT*^ overexpressing cells confirming that the events activated by HSPB8 are specific and generated by the protein in its native form (Fig. 5g, h).

### Effect of HSPB8 overexpression on autophagy in BLM and A375 cells

**Fig. 6:**
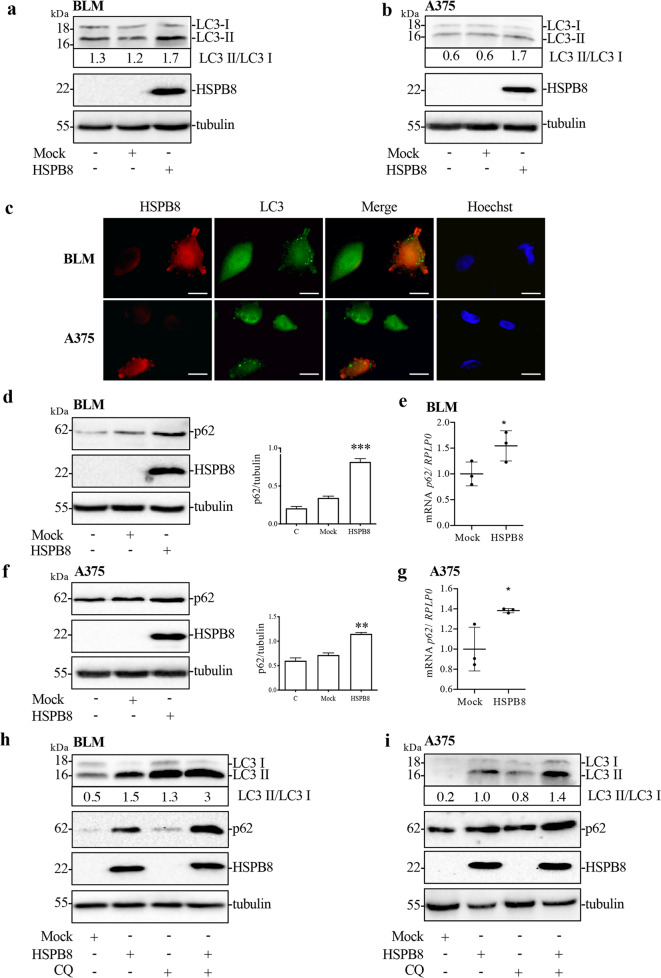
Effect of HSPB8 overexpression on autophagy in BLM and A375 cells. **a**, **b** WB analysis of LC3 in BLM (**a**) and A375 (**b**) overexpressing HSPB8 (48 h). **c** LC3 puncta (green) and HSPB8 expression (red) were carried out by immunofluorescence in BLM and A375 cells overexpressing HSPB8 (48 h). Nuclei were stained by Hoechst. Scale bar, 20 μm. **d**, **f** Protein expression of p62 in BLM (**d**) and A375 (**f**) cells overexpressing HSPB8 (48 h). The quantification results were calculated over three individual experiments. Bar graph represents the mean optical density ± SD. Statistical analysis was performed using One-way ANOVA followed by Bonferroni post-hoc test (**p < 0.01 vs. Control (C) and mock; ***p < 0.001 vs. Control (C) and mock). **e**, **g** *p62* mRNA levels in BLM (**e**) and A375 (**g**) cells overexpressing HSPB8 (24 h). Data have been normalized to the amount of *RPLP0* mRNA. Bar graph represents the mean of three independent biological samples (*N* = 3) ±SD. Statistical analysis was performed using Student’s *t*-test (*p < 0.05 vs. mock). **h**, **i** WB analysis of LC3 and p62 in BLM (**h**) and A375 (**i**) cells overexpressing HSPB8 (48 h) treated with CQ 10 μM for the last 24 h of transfection.

It is known that HSPB8, together with its co-chaperone BAG3, exerts a pro-autophagic activity [18]. We therefore evaluated a possible autophagy activation following HSPB8 overexpression in BLM and A375 cells. HSPB8 overexpression increased LC3-II / LC3-I ratio (Fig. 6a, b) and LC3 puncta (Fig. 6c) in both cell lines. HSPB8 overexpression also increased the SQSTM1/p62 (p62) protein and mRNA levels in both BLM (Fig. 6d, e) and A375 cells (Fig. 6f, g). We therefore analyzed whether the accumulation of p62 was due to an impairment of autophagic flux, testing LC3 and p62 protein levels after HSPB8 overexpression in the presence of chloroquine (CQ, an inhibitor of lysosomal activity). In both cell lines, CQ treatment of HSPB8 overexpressing cells resulted in an increase of LC3-II and p62 levels suggesting that the autophagic flux was not impaired by HSPB8 overexpression (Fig. 6h, i).

### Involvement of HSPB8-induced autophagy in BLM and A375 cell proliferation

**Fig. 7:**
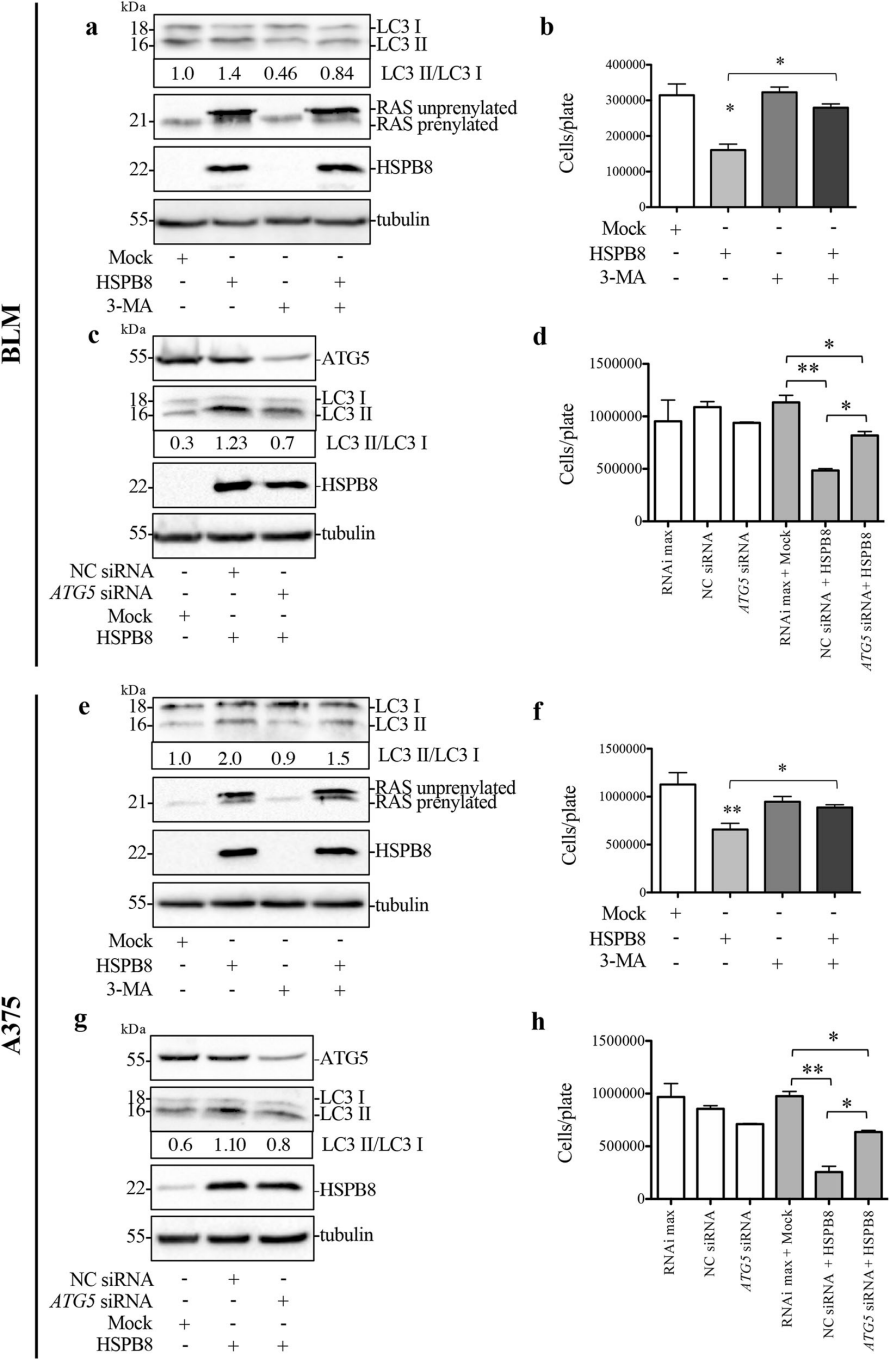
Involvement of HSPB8-induced autophagy in BLM and A375 cell proliferation. **a**, **b** BLM cells were pre-treated with or without 1 mM 3-MA for 1 h before HSPB8 overexpression (48 h). WB analysis of LC3 and RAS protein were carried out (**a**) and the effect on cell proliferation was evaluated by cell count (**b**). Data are mean ± SD of four independent biological samples (*N* = 4). Each experiment was repeated three times. Statistical analysis was performed using One-way ANOVA followed by Bonferroni post-hoc test; (*p < 0.05). **c** WB analysis of ATG5 and LC3 in BLM cells transfected with 20 nM negative control siRNA (NC) or *ATG5* siRNA for 72 h and HSPB8 overexpression (48 h). **d** Effect of *ATG5* siRNA on proliferation of BLM cells overexpressing HSPB8. Data are mean ± SD of four independent biological samples (*N* = 4). Each experiment was repeated three times. Statistical analysis was performed using One-way ANOVA followed by Bonferroni post-hoc test; (*p < 0.05; **p < 0.01). **e**, **f** A375 cells were pre-treated with or without 1 mM 3-MA for 1 h before HSPB8 overexpression (48 h). At the end of the treatment WB analysis of LC3 and RAS protein were carried out (**e**) and the effect on cell proliferation was evaluated by cell count (**f**). Four independent biological samples for each condition were analyzed (*N* = 4), bar graph represents the mean relative cell viability ± SD. Each experiment was repeated three times. Statistical analysis was performed using One-way ANOVA followed by Bonferroni post-hoc test (*p < 0.05, **p < 0.01). **g** WB analysis of ATG5 and LC3 in A375 cells transfected with 20 nM negative control siRNA (NC) or *ATG5* siRNA for 72 h and HSPB8 overexpression (48 h). **h** Effect of *ATG5* siRNA on cell proliferation of A375 cells overexpressing HSPB8. Data are mean ± SD of four independent biological samples (*N* = 4). Each experiment was repeated three times. Statistical analysis was performed using One-way ANOVA followed by Bonferroni post-hoc test; (*p < 0.05; **p < 0.01).

Further, to examine if autophagy activation is involved in HSPB8 antitumoral activity we analyzed the effect of HSPB8 overexpression in presence of 3-Methyladenine (3-MA, an inhibitor of the early steps of the autophagic process). We found that 3-MA blocked the autophagic process (Fig. 7a) and counteracted the antiproliferative effect induced by HSPB8 overexpression in BLM cells (Fig. 7b).

To clarify the functional relationship between autophagic activation and HSPB8-induced RAS-prenylation inhibition, we evaluated the effect of 3-MA on RAS expression demonstrating that autophagy inhibition does not modify the effect of HSPB8 on unprenylated form of RAS (Fig. 7a).

Since 3-MA could also inhibit other signaling pathways, we analyzed the role of autophagy on the HSPB8 antiproliferative effect downregulating the expression of autophagic gene *ATG5*.

We performed *ATG5* silencing for 72 h followed by HSPB8 overexpression in the last 48 h.

WBs shown in Fig. 7c confirm the HSPB8 overexpression and a significant reduction of ATG5 protein levels in presence of *ATG5* siRNA. The autophagy inhibition was confirmed by LC3-II/LC3-I decrease (Fig. 7c).

The *ATG5* siRNA counteracted the antiproliferative effect induced by HSPB8 overexpression in BLM cells (Fig. 7d).

Overlapping results were obtained in A375 cell line (Fig. 7e–h).

These results suggest that the autophagy induced by HSPB8 overexpression is a crucial cellular process for cell proliferation regulation and that its inhibition counteracts the antitumoral role of HSPB8 overexpression in melanoma cells.

## Discussion

In recent years, several studies focused on the role of HSPB8 in tumors, with conflicting results. Some studies demonstrated that HSPB8 is highly expressed in breast cancers [46, 47, 50–55], myeloma [56], ovarian cancer [57], gastric cancer [58], lung cancer [59], and cholangiocarcinoma [60] where it increases cell proliferation, migration and tumorigenic activity. Conversely, other studies showed that HSPB8 is downregulated in leukemia and lymphoma [61], glioblastoma [62, 63], hepatocarcinoma [64, 65], prostate cancer [39, 66, 67], melanoma [39, 40, 68–70] and its upregulation contributes to induce cell death and chemosensitivity generating an overall antitumoral effect.

The aim of this work was therefore to better clarify the role of HSPB8 in human melanoma with specific mutational status.

Our studies then focused on melanoma cells carrying the Q61R *NRAS* mutation (BLM cells) or the V600E *BRAF* mutation (A375 cells), representing the main mutations identified in melanoma patients. Both cell types express very low HSPB8 levels, and restoration of HSPB8 expression exerts an antiproliferative and cytotoxic activity independently of the mutation characterizing the two cell lines analyzed. These data are in line with previous studies [39, 40, 68–70] suggesting that HSPB8 acts as tumor suppressor in melanoma and its downregulation may promotes melanoma tumorigenesis.

Also, the studies of EMT-like reversion and cellular migration, the first event of an invasive process, confirmed the antitumoral effects of HSPB8 in melanoma. In fact, while HSPB8 exerts a pro-migratory ability in breast cancer [46], intrahepatic cholangiocarcinoma [60] and ovarian cancer [57], in which HSPB8 plays a pro-tumoral role, we found that HSPB8 reduces the migratory properties of both cell lines analyzed highlighting how this protein can reduce the metastatic potential of melanomas with different mutational status. Thus, it is likely that the loss of the HSPB8 protection contributes to the development of metastatic melanoma. We thus analyzed the molecular mechanism associated to HSPB8 antitumoral action, focusing on the signal transduction pathways constitutively activated by the specific mutations present in the cell lines used. With this approach, we clearly demonstrated the existence of a link between HSPB8 and the prenylation status of RAS. Indeed, in BLM cells, we evaluated the impact of HSPB8 on NRAS expression and found that its overexpression induces a biochemical modification of RAS compatible with a change in its prenylation. Notably, prenylation induces the RAS protein to shuttle from cytoplasm to the plasma membrane where it acquires its active status. Conversely, the unprenylated RAS accumulates in the cytoplasm in its inactive status. Indeed, the decreased expression of RAS in BLM membrane preparations after overexpression of HSPB8 confirms the ability of HSPB8 to reduce the active form of RAS. Overlapping results were obtained in A375 cells where the expression of BRAF^V600E^ was not modified by the overexpression of HSPB8.

The role of prenylation on RAS activity is well characterized and many anticancer molecules exploit the ability to inhibit this process [71]. Several studies proved that the inhibition of RAS-prenylation regulates tumoral growth and invasion [72, 73], while RAS de-prenylation overcomes drug resistance [74]. On these bases, in melanoma, the inhibition of RAS-prenylation has been suggested as a possible therapy in *NRAS*-mutant tumors [75]. Therefore, our results demonstrate the relevance of HSPB8 in counteracting melanoma aggressiveness, via a decrease in RAS-prenylation that reduces the activity of this protein.

We also demonstrated that the activation of the transduction pathways regulated by the specific mutations characterizing our cellular models is modified by HSPB8. In fact, we found that HSPB8 overexpression counteracts the ERK phosphorylation, inhibiting the MAPK pathway and reducing cyclin D expression in BLM cells. Also, the Akt/mTOR pathway is controlled by HSPB8 leading to a reduction of Akt and mTOR phosphorylation. Conversely, only Akt and mTOR phosphorylation were inhibited in A375 cells, demonstrating that HSPB8 is unable to counteract the effects of the constitutive activation of mutated BRAF. These results confirm that HSPB8 regulates RAS activity both in *NRAS*-mutant and *NRAS*-wild-type melanoma cells.

Of interest, these data are in line with those obtained in other type of cancers, demonstrating that HSPB8 repressed hepatocarcinoma progression and migration by downregulation of PI3K/Akt signaling pathway [64].

The overexpression of mutated HSPB8 (HSPB8^K141E^ and HSPB8^K141N^) in both cell lines abolished HSPB8 ability to induce RAS de-prenylation, Akt dephosphorylation and the protective antiproliferative activity. This confirms that HSPB8 is active in its native conformation, while the HSPB8 mutations, that determine a protein structure modification, abrogate this activity. This phenomenon could be due to the inability of HSPB8 to recruit the molecular co-chaperones such as BAG3, an hypothesis that find support in the loss of function of HSPB8^K141E^ and HSPB8^K141N^ [76].

It is known that HSPB8 is involved in autophagy [18, 77], and that autophagy is deeply involved in tumorigenesis and cancer progression. In the early stage of cancer progression, autophagy preserves cell survival through cellular catabolism. During the late stage of tumorigenesis autophagy is hyperactivated as reported by numerous studies describing that dysfunctional autophagy is associated with several pathophysiological processes, including cancer [78].

Indeed, autophagy can either activate cell survival or trigger cell death of tumor cells [79]. Our results demonstrated that HSPB8 overexpression promotes the autophagic flux both in BLM and A375 cells. Notably, the autophagy inhibitor 3-MA and genetic inhibition of autophagy by *ATG-5* silencing counteracted HSPB8 antiproliferative activity, demonstrating that HSPB8-activated autophagy exerts an antitumoral action.

**Fig. 8:**
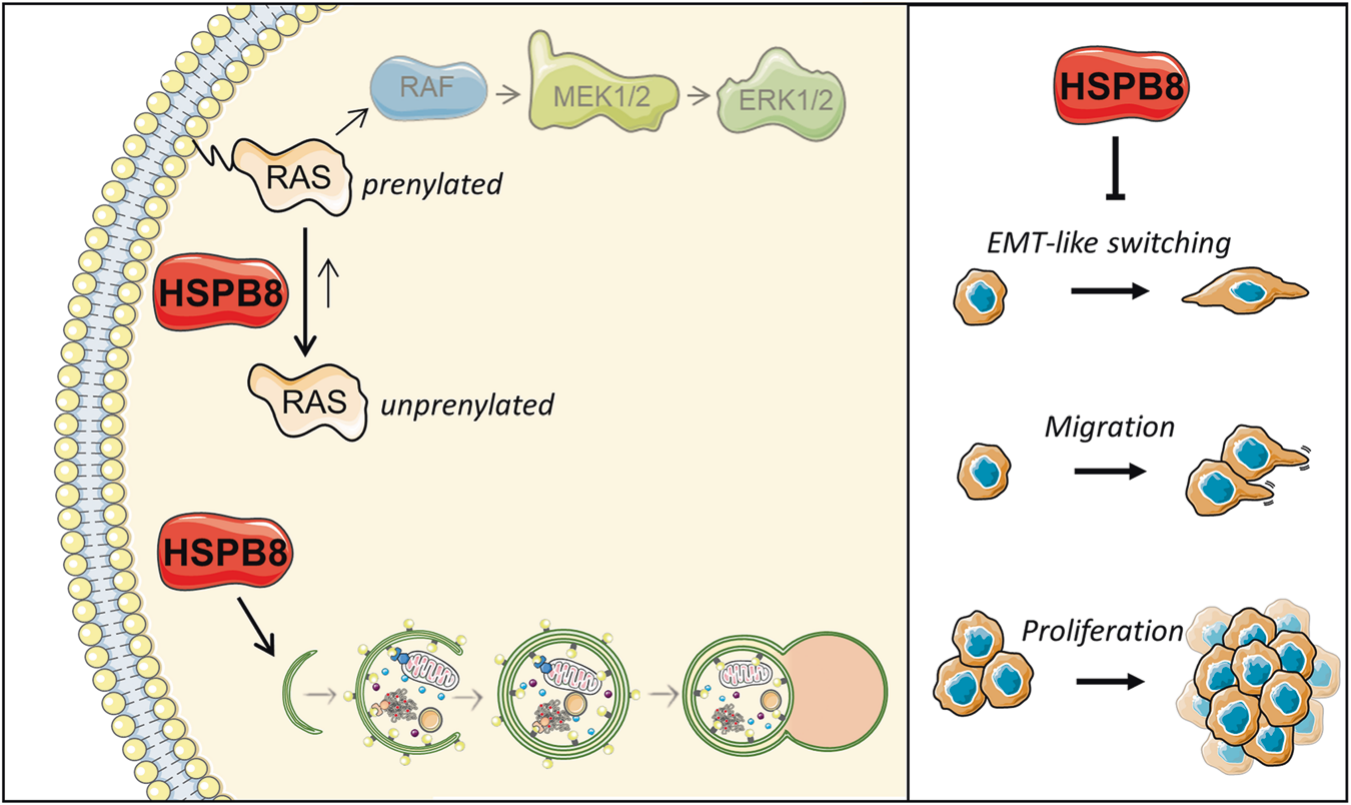
Mechanisms at the basis of the antitumoral activity of HSPB8 in melanomas cell lines. When expressed in melanoma cell lines, HSPB8 favors the unprenylated form of RAS over the prenylated form, inhibiting the RAF/MEK/ERK pathway, and facilitates autophagy. Therefore, HSPB8 exerts an antitumoral activity by inhibiting (i) the epithelial-mesenchymal transition (EMT)-like switching, (ii) migration, and (iii) proliferation. This figure was partly generated using Servier Medical Art templates, provided by Servier, licensed under a Creative Commons Attribution 3.0 Unported License; https://smart.servier.com.

In conclusion, in melanoma HSPB8 counteracts proliferative and migratory cellular phenotype by enhancing inactive unprenylated RAS and autophagy (Fig. 8). Thus, the HSPB8 expression might represent a promising factor for the onset and the progression of melanoma.

## Supplementary information

Supplementary material

## Data Availability

All data generated during this study are included in this published article and its supplementary files. The raw data analyzed during the current study are available from the corresponding author on reasonable request.
