# Supplementary material for: HSPB8 counteracts tumor activity of BRAF- and NRAS-mutant melanoma cells by modulation of RAS-prenylation and autophagy
Source: Cell Death Dis. 2022 Nov 18;13(11):973. doi: 10.1038/s41419-022-05365-9 (PMC9674643; doi:10.1038/s41419-022-05365-9)

Supplementary 1

Fig. 1b

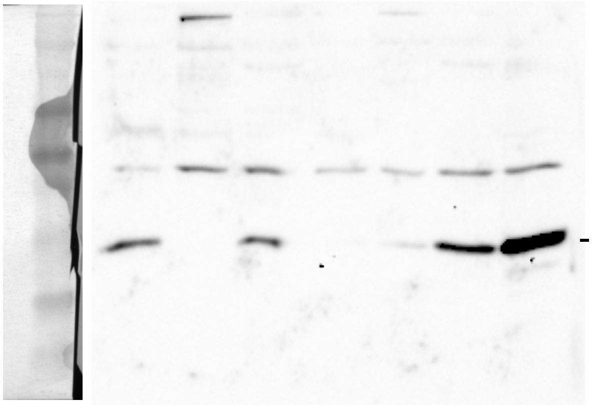

Fig. 1d

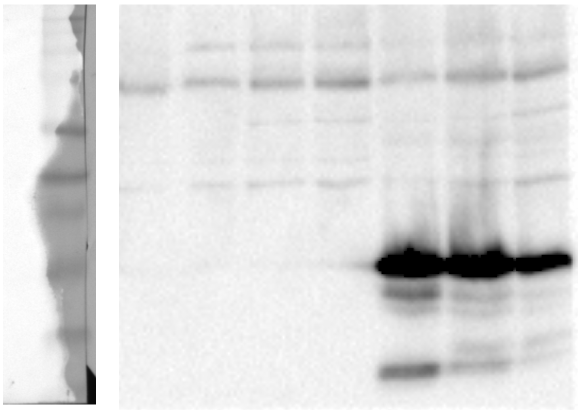

Fig. 1e

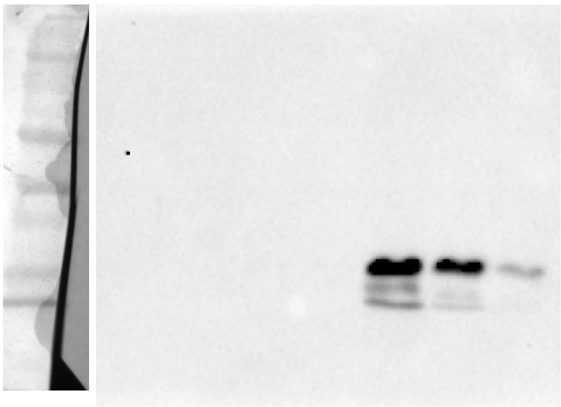

Supplementary 2

Fig. 2c

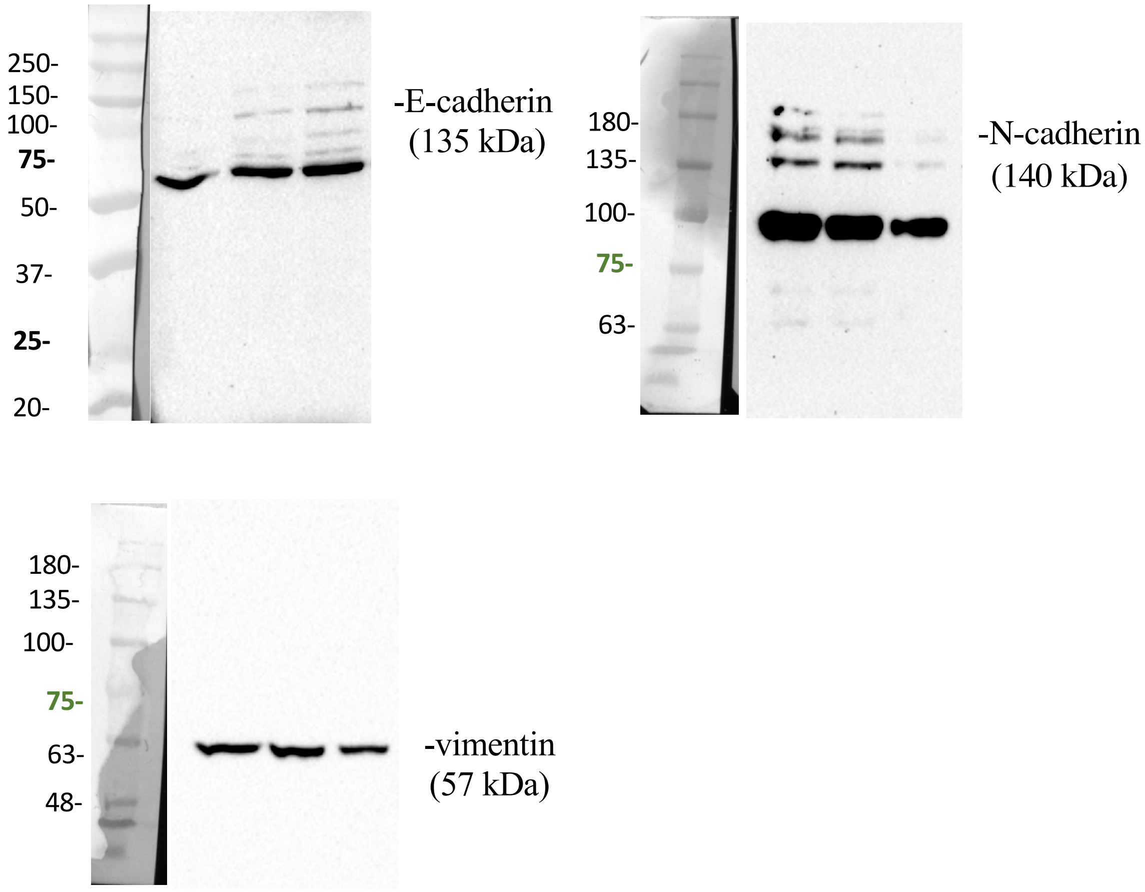

Fig. 2d

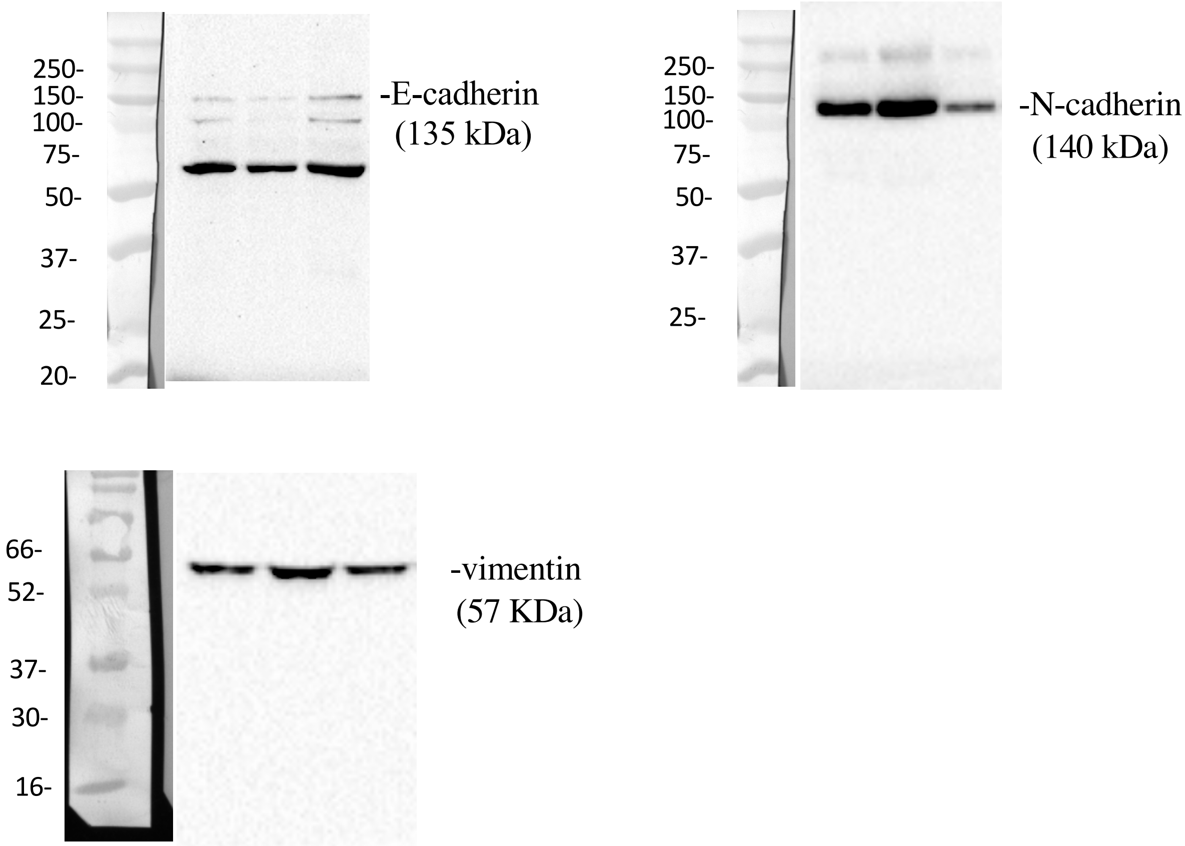

Supplementary 3

Fig. 3a

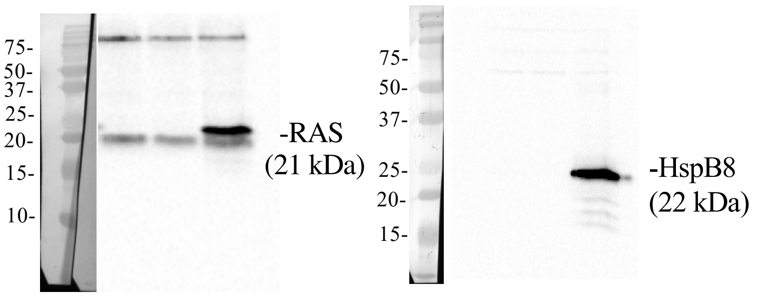

Fig. 3b

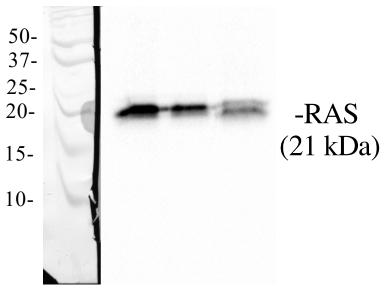

Fig. 3c

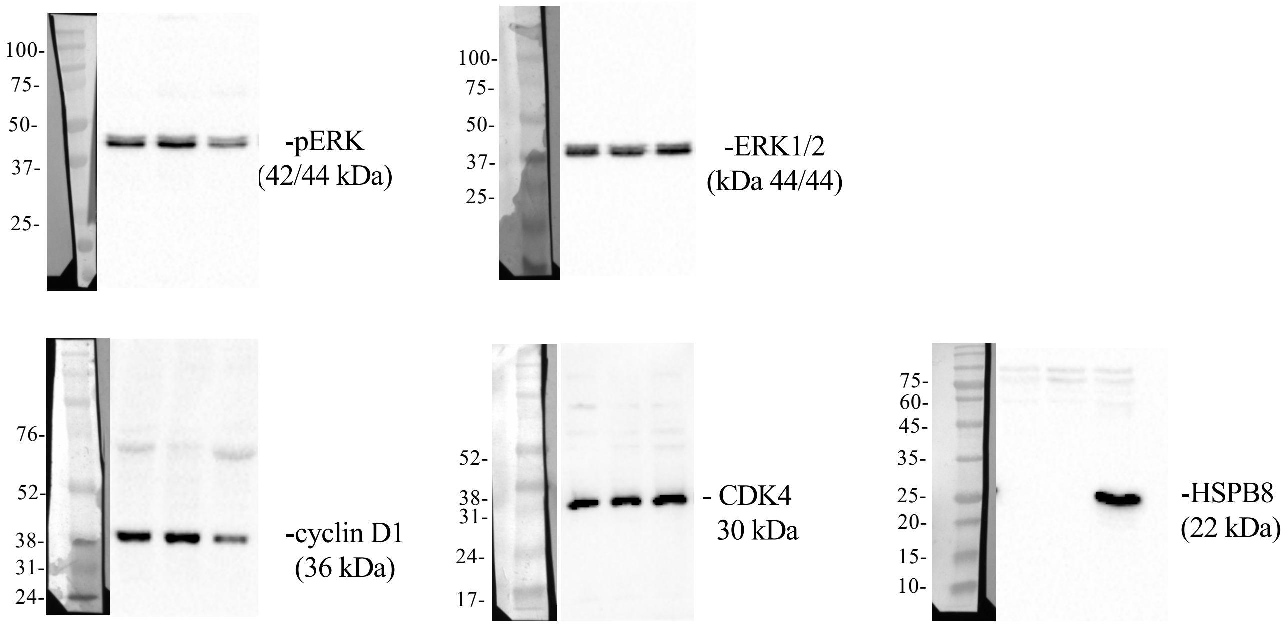

Fig. 3d

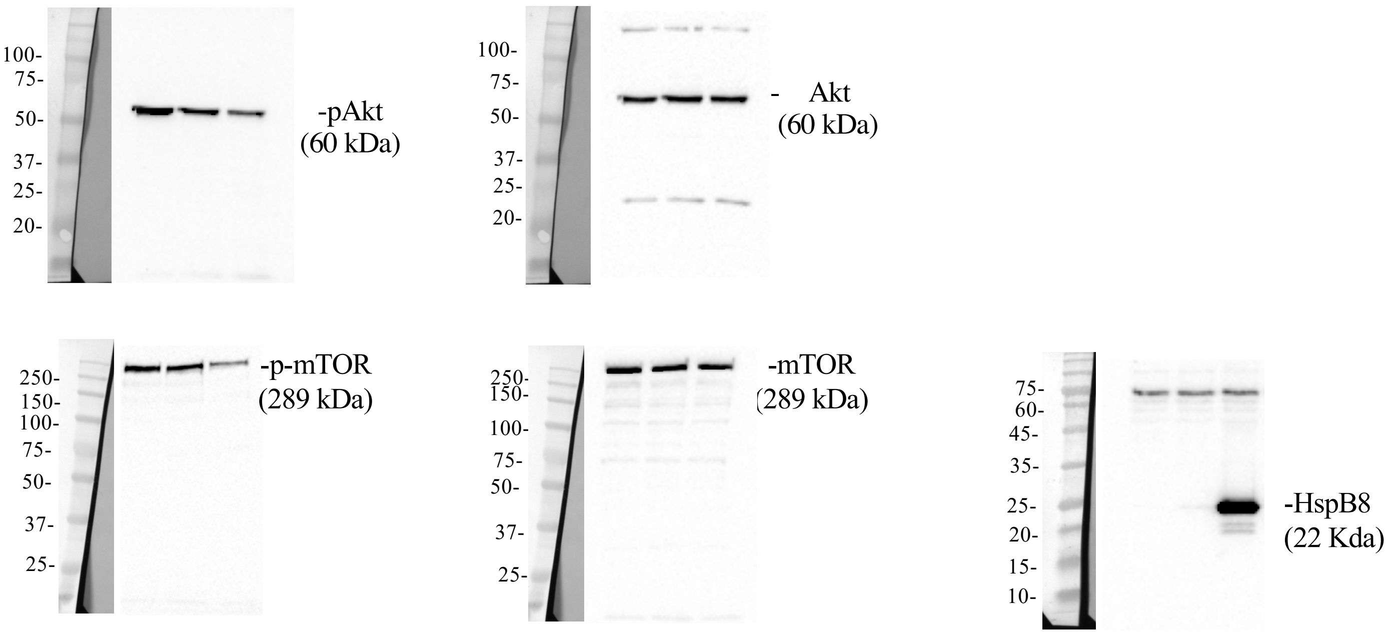

Supplementary 4

Fig. 4a

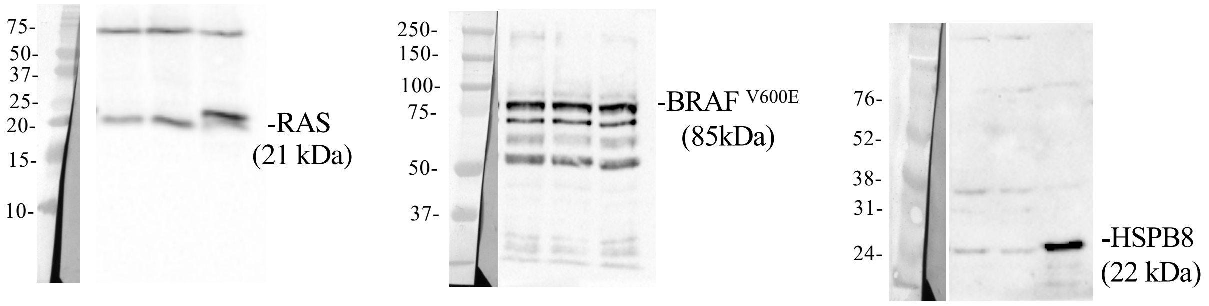

Fig. 4b

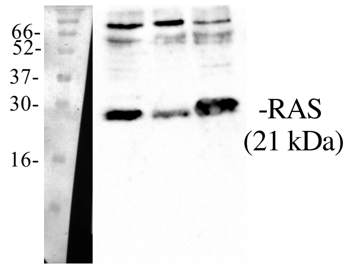

Fig. 4c

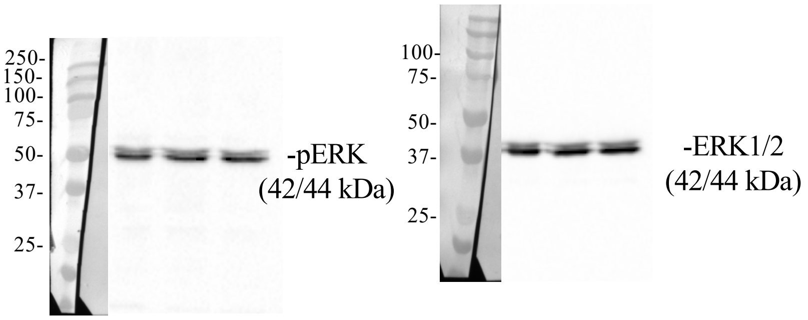

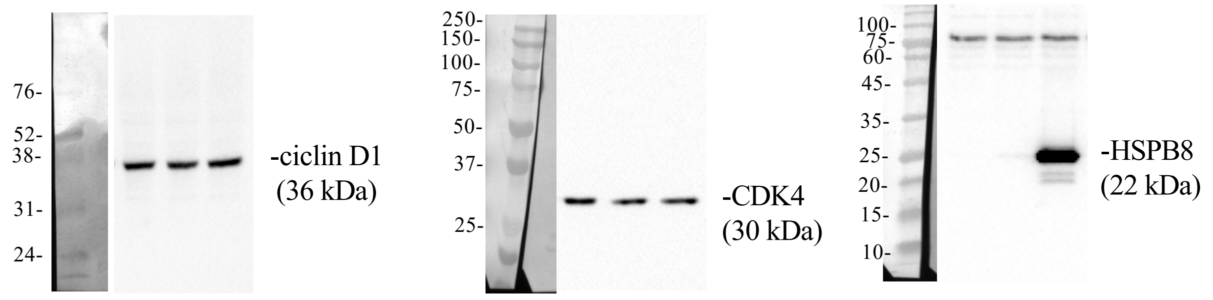

Fig. 4d

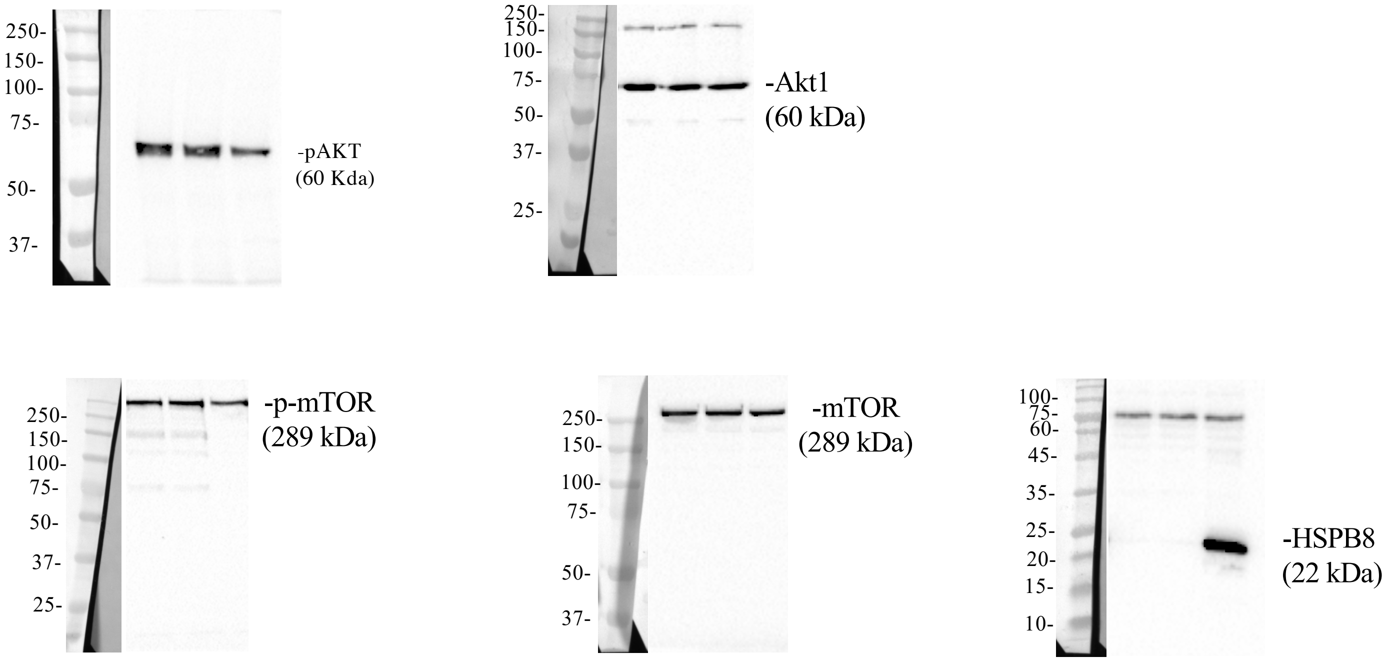

Supplementary 5

Fig. 5a

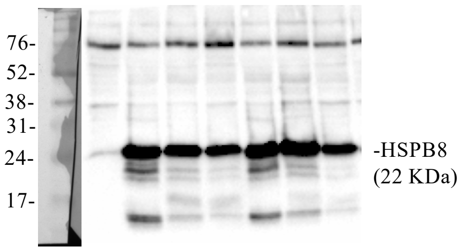

Fig. 5b

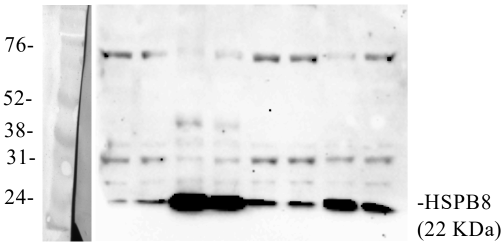

Fig. 5c

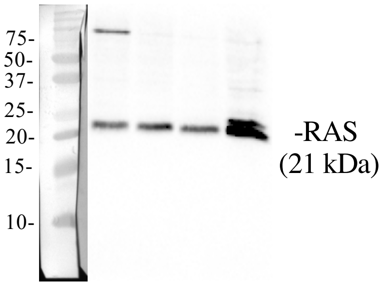

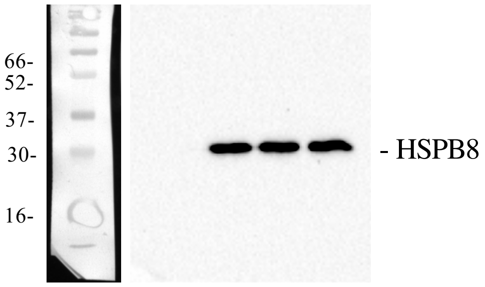

Fig. 5d

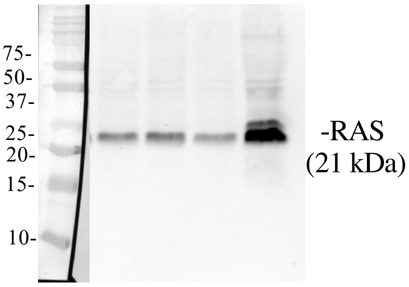

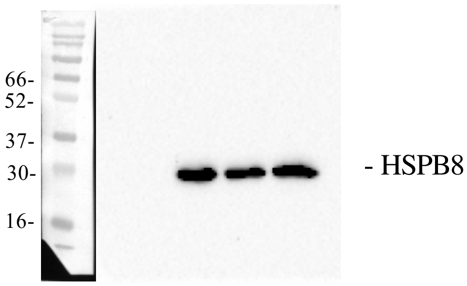

Fig. 5e

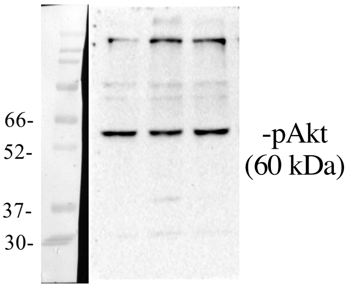

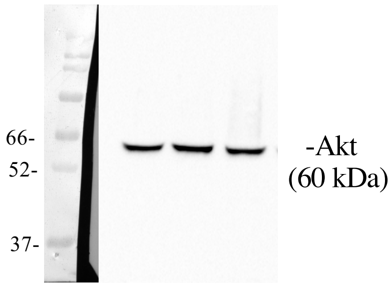

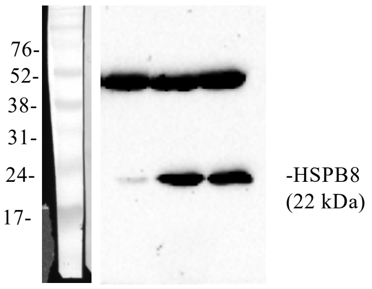

Fig. 5f

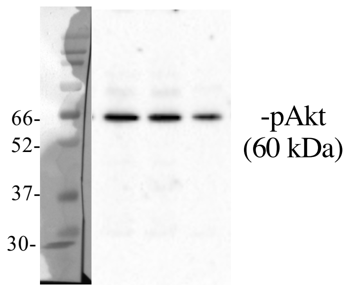

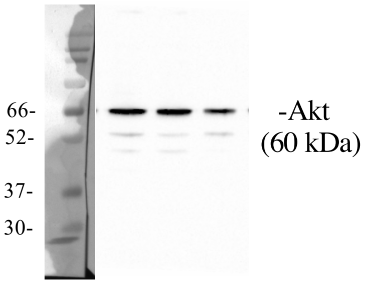

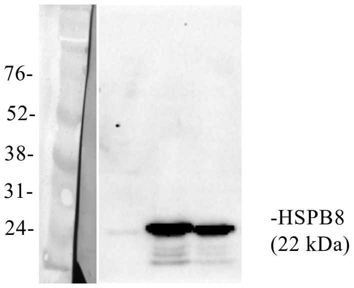

Supplementary 6

Fig. 6a

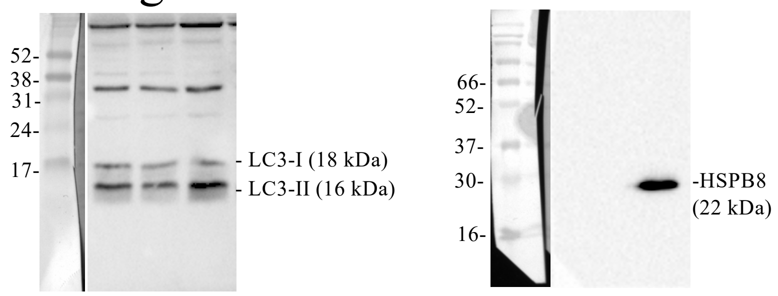

Fig. 6b

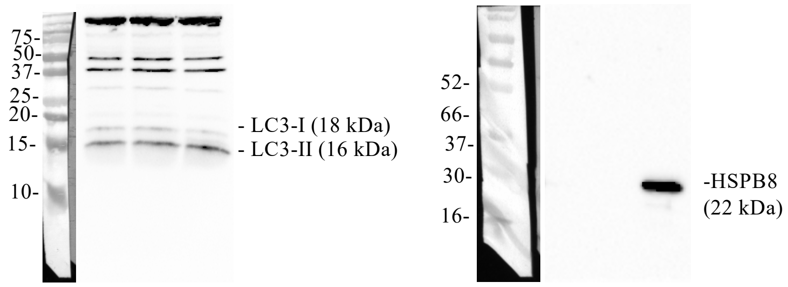

Fig. 6d

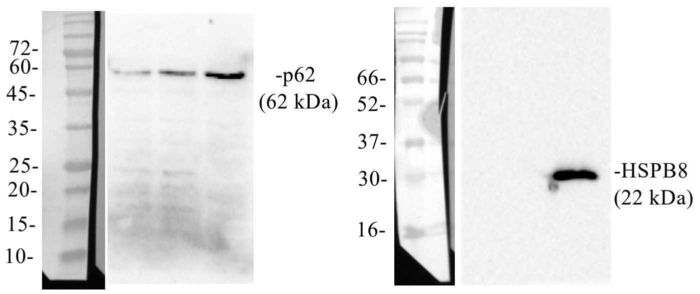

Fig. 6f

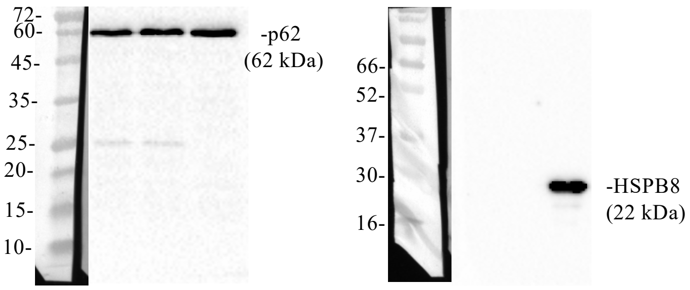

Fig. 6h

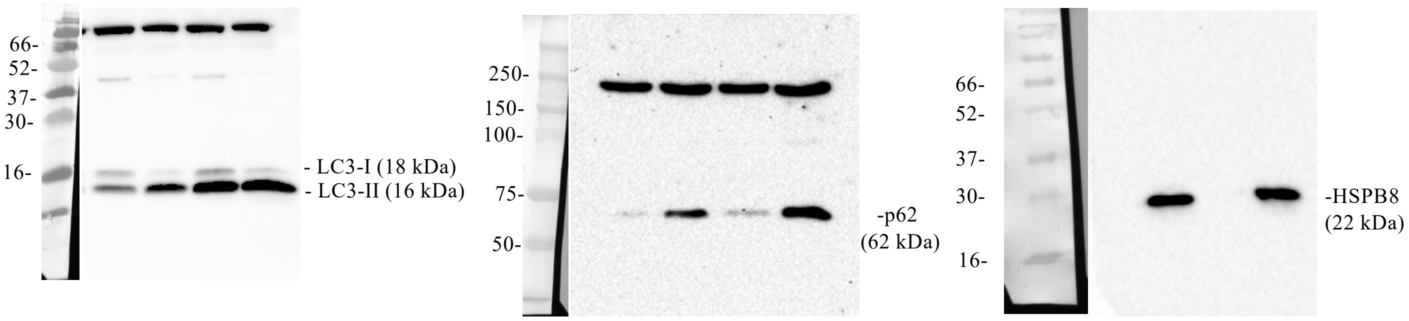

Fig. 6i

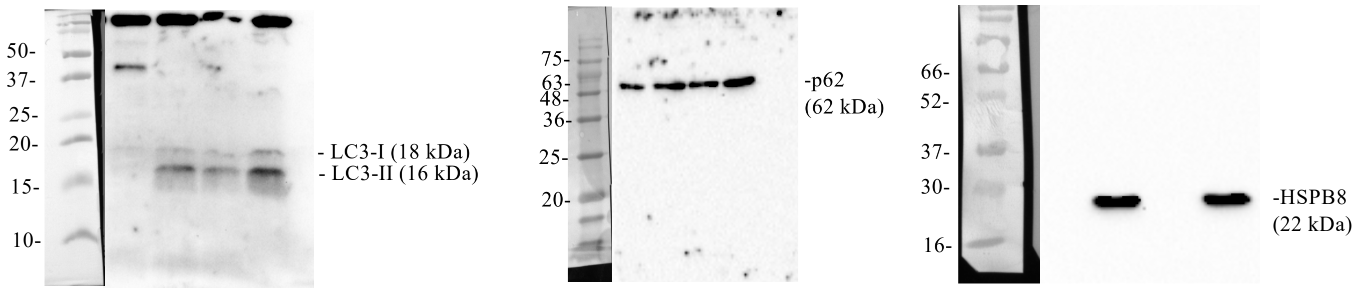

Supplementary 7

Fig. 7a

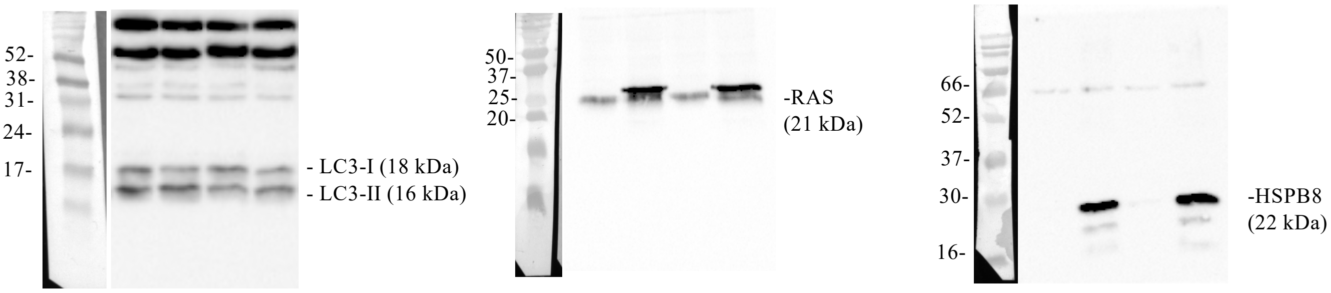

Fig. 7c

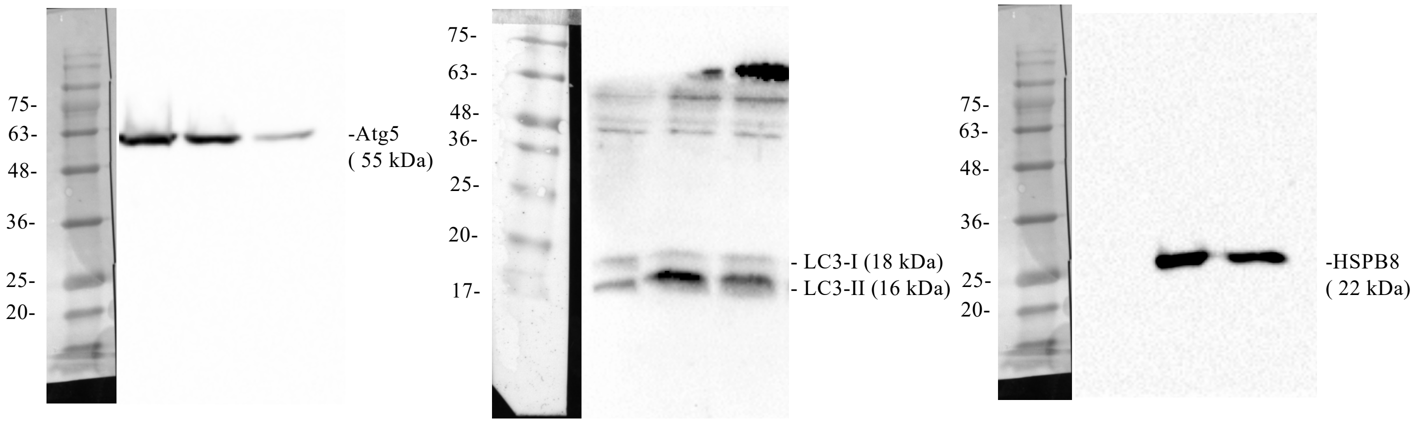

Fig.7e

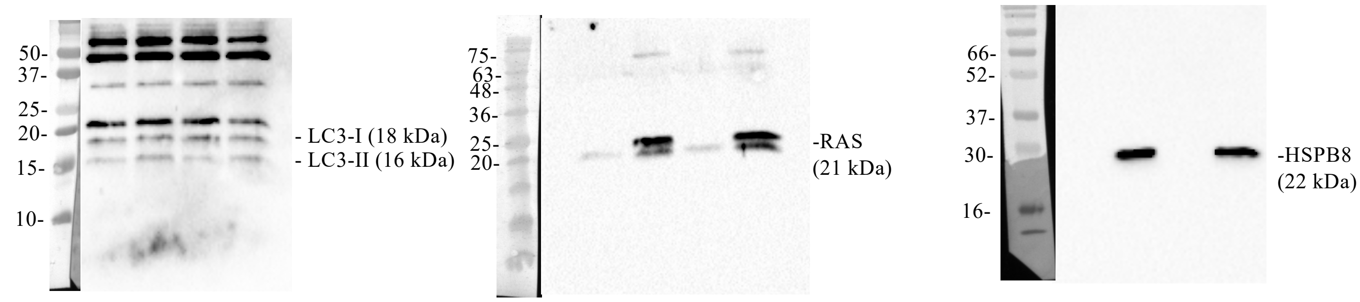

Fig.7g

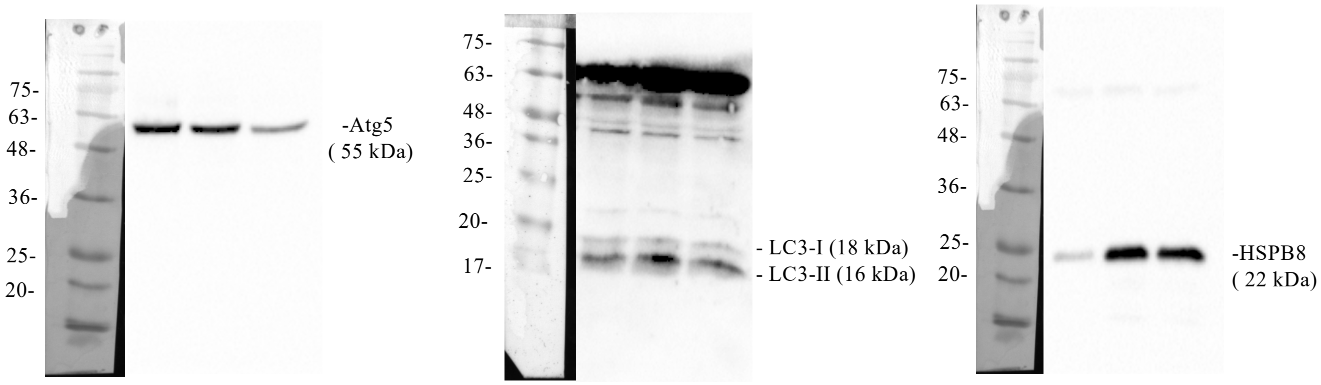

Supplement: Supplementary file 1 — Supplementary material [file 41419_2022_5365_MOESM1_ESM.pdf]
